# Changing patterns in reporting and sharing of review data in systematic reviews with meta-analysis of the effects of interventions: cross sectional meta-research study

**DOI:** 10.1136/bmj-2022-072428

**Published:** 2022-11-22

**Authors:** Phi-Yen Nguyen, Raju Kanukula, Joanne E McKenzie, Zainab Alqaidoom, Sue E Brennan, Neal R Haddaway, Daniel G Hamilton, Sathya Karunananthan, Steve McDonald, David Moher, Shinichi Nakagawa, David Nunan, Peter Tugwell, Vivian A Welch, Matthew J Page

**Affiliations:** 1 Methods in Evidence Synthesis Unit, School of Public Health and Preventive Medicine, Monash University, Melbourne, VIC, Australia; 2 Leibniz-Centre for Agricultural Landscape Research, Müncheberg, Germany; 3 Stockholm Environment Institute, Stockholm, Sweden; 4 African Centre for Evidence, University of Johannesburg, Johannesburg, South Africa; 5 School of BioSciences, University of Melbourne, Melbourne, VIC, Australia; 6 Interdisciplinary School of Health Sciences, University of Ottawa, Ottawa, ON, Canada; 7 Bruyère Research Institute, Ottawa, ON, Canada; 8 Centre for Journalology, Clinical Epidemiology Programme, Ottawa Hospital Research Institute, Ottawa, ON, Canada; 9 School of Epidemiology and Public Health, Faculty of Medicine, University of Ottawa, Ottawa, ON, Canada; 10 Evolution & Ecology Research Centre and School of Biological, Earth and Environmental Sciences, University of New South Wales, Sydney, NSW, Australia; 11 Centre for Evidence-Based Medicine, Nuffield Department of Primary Care Health Sciences, Oxford University, Oxford, UK; 12 Department of Medicine, Faculty of Medicine, University of Ottawa, Ottawa, ON, Canada

## Abstract

**Objectives:**

To examine changes in completeness of reporting and frequency of sharing data, analytical code, and other review materials in systematic reviews over time; and factors associated with these changes.

**Design:**

Cross sectional meta-research study.

**Population:**

Random sample of 300 systematic reviews with meta-analysis of aggregate data on the effects of a health, social, behavioural, or educational intervention. Reviews were indexed in PubMed, Science Citation Index, Social Sciences Citation Index, Scopus, and Education Collection in November 2020.

**Main outcome measures:**

The extent of complete reporting and the frequency of sharing review materials in the systematic reviews indexed in 2020 were compared with 110 systematic reviews indexed in February 2014. Associations between completeness of reporting and various factors (eg, self-reported use of reporting guidelines, journal policies on data sharing) were examined by calculating risk ratios and 95% confidence intervals.

**Results:**

Several items were reported suboptimally among 300 systematic reviews from 2020, such as a registration record for the review (n=113; 38%), a full search strategy for at least one database (n=214; 71%), methods used to assess risk of bias (n=185; 62%), methods used to prepare data for meta-analysis (n=101; 34%), and source of funding for the review (n=215; 72%). Only a few items not already reported at a high frequency in 2014 were reported more frequently in 2020. No evidence indicated that reviews using a reporting guideline were more completely reported than reviews not using a guideline. Reviews published in 2020 in journals that mandated either data sharing or inclusion of data availability statements were more likely to share their review materials (eg, data, code files) than reviews in journals without such mandates (16/87 (18%) *v* 4/213 (2%)).

**Conclusion:**

Incomplete reporting of several recommended items for systematic reviews persists, even in reviews that claim to have followed a reporting guideline. Journal policies on data sharing might encourage sharing of review materials.

## Introduction

Systematic reviews underpin many government policies and professional society guideline recommendations.^1^ To ensure systematic reviews are valuable to decision makers, authors should report the complete methods and results of their review. Complete reporting allows readersto judge whether the chosen methods could have biased the review findings. Incomplete reporting of the methods prevents such an assessment and can preclude attempts to replicate the findings. Several meta-research studies have evaluated the completeness of the reporting of methods and results in systematic reviews and meta-analyses. Many of these studies were narrow in scope, focusing only on reviews of specific health topics^2^ ^3^ ^4^ ^5^ ^6^ or reviews published in selected journals.^7^ ^8^ In other studies, the sample of reviews examined was more diverse, but contained reviews published almost a decade ago^9^ ^10^ or was evaluated against a small set of reporting items,^1^ meaning that comprehensive data on the current state of the reporting of systematic reviews are lacking.

To resolve incomplete reporting of methods and results in systematic reviews, several reporting guidelines have been developed, with the PRISMA (Preferred Reporting Items for Systematic reviews and Meta-Analyses) statement^11^ being among the more widely used.^12^ Reporting guidelines provide a structure for reporting a systematic review, along with recommendations of items to report.^13^ Originally released in 2009, PRISMA was recently updated (to PRISMA 2020) to reflect advances in systematic review methodology.^14^ The few studies examining the impact of PRISMA suggest that some items (eg, inclusion of a flow diagram) improved after its introduction, but that others (eg, mention of a review protocol) remained infrequently reported.^15^ These analyses are limited to reviews published before 2015, and therefore the influence of reporting guidelines on more recent systematic reviews is unclear.

In addition to transparent reporting, advocates for research transparency^16^ ^17^ also recommend that authors share systematic review data files and analytical code used to generate meta-analyses.^18^ While all data for a meta-analysis are typically summarised in tables or forest plots, sharing an editable file containing extracted data (eg, CSV, RevMan (.rm5)) reduces the time and risk of errors associated with manual extraction of such data. This then facilitates the review’s reuse in future updates and replications, or its inclusion in overviews of reviews, clinical practice guidelines, educational materials, and meta-research studies.^18^ ^19^ Sharing review data files is relatively easier than sharing individual participant data from primary studies, and signals that review authors are committed to practices that they encourage from authors of primary studies, who are often asked to share their data. Infrequent sharing of data in systematic reviews in health research has been observed, but these findings might not be generalisable to all health topics^4^ or across journals.^7^ Moreover, the types of data shared (eg, unprocessed data extracted from reports, data included in meta-analyses) has not been examined, nor has the impact of journals’ data sharing policies on rates of sharing in systematic reviews.

Without a current, comprehensive evaluation of the completeness of reporting of systematic reviews, we lack data on which items are infrequently reported and require most attention from authors, peer reviewers, editors, and educators. Furthermore, without data on the frequency and type of materials review authors currently share, we lack insight into how receptive review authors are to calls to share data underlying research projects. To review these research gaps, we aimed to:

- Evaluate the completeness of reporting in a cross section of systematic reviews with meta-analysis published in 2020
- Evaluate the frequency of sharing review data, analytical code, and other materials in the same cohort of reviews
- Compare reporting in these reviews with a sample of reviews published in 2014;
- Investigate the impact of reporting guidelines on the completeness of reporting in reviews published in 2020
- Investigate the impact of journals’ data sharing policies on the frequency of data sharing in reviews published in 2020.

We chose 2014 as the year against which to compare reviews from 2020 because we had access to the raw data on completeness of reporting in a sample of reviews from 2014^10^ that met the same eligibility criteria and were evaluated using similar methods as the reviews sampled from 2020.

## Methods

This study was conducted as one of a group of studies in the REPRISE (REProducibility and Replicability In Syntheses of Evidence) project. The REPRISE project is investigating various aspects relating to the transparency, reproducibility, and replicability of systematic reviews with meta-analysis of the effects of health, social, behavioural, and educational interventions. Methods for all studies were prespecified in the same protocol.^20^ Deviations from the protocol for the current study are outlined in the supplemental data.

### Identification and selection of articles

We included a random sample of systematic reviews with meta-analysis of the effects of a health, social, behavioural, or educational intervention (ie, any intervention designed to improve health (defined as “a state of complete physical, mental and social well-being and not merely the absence of disease or infirmity”^21^),promote social welfare and justice, change behaviour, or improve educational outcomes; see the supplemental data for full eligibility criteria). To be considered a systematic review, authors needed to have, at a minimum, clearly stated their review objective(s) or question(s); reported the source(s) (eg, bibliographic databases) used to identify studies meeting the eligibility criteria; and reported conducting an assessment of the validity of the findings of the studies included (eg, via an assessment of risk of bias or methodological quality). We did not exclude systematic reviews providing limited detail about the methods used. We only included systematic reviews that presented results for at least one pairwise meta-analysis of aggregate data. Systematic reviews with network meta-analyses were eligible if they included at least one direct (ie, pairwise) comparison that fulfilled the criteria mentioned above. Systematic reviews with only meta-analyses of individual participant data were excluded because all eligible systematic reviews in this study will be subjected to a reproducibility check in another component of the REPRISE project,^20^ and we lacked the resources to reproduce these meta-analyses of individual participant data. Furthermore, only reviews written in English were included.

Using search strategies created by an information specialist (SM), we systematically searched PubMed, Science Citation Index, and Social Sciences Citation Index via Web of Science, Scopus via Elsevier, and Education Collection via ProQuest for systematic reviews indexed from 2 November to 2 December 2020. All searches were conducted on 3 December 2020. An example of the search strategy for PubMed was (meta-analysis[PT] OR meta-analysis[TI] OR systematic[sb]) AND 2020/11/02:2020/12/02[EDAT]). Search strategies for all databases are available in the supplemental data.

We used Endnote version 9.3.3 for automatic deduplication of records, then randomly sorted unique records in Microsoft Excel using the RAND() function, and imported the first 2000 records yielded from the search into Covidence^22^ for screening. Two authors (MJP and either P-YN or RK) independently screened the titles and abstracts of the 2000 records against the eligibility criteria. We retrieved the full text of all records deemed potentially eligible, and two authors (P-YN and either MJP or RK) independently evaluated them in random order against the eligibility criteria until we reached our target sample size of 300 systematic reviews. Any disagreement at each stage of screening was resolved via discussion or adjudication by the senior reviewer (MJP). Because this study was primarily descriptive, we aimed to examine reporting across a range of practices. We selected our sample size of 300 systematic reviews as a balance of feasibility and precision. This sample size allowed us to restrict the width of a 95% two sided Wald type confidence interval around the estimated percentage of reviews reporting a particular practice to a maximum of 6%, assuming a prevalence of 50%. For a prevalence of less (or greater) than 50%, the absolute width will be smaller. This maximum confidence interval width was small enough such that our interpretation of the confidence interval limits would be generally consistent.

### Data collection

Two authors (PN and either MJP, RK, or ZA) collected data independently and in duplicate from all of the 300 systematic reviews using a standardised form created in REDCap version 10.6.12, hosted at Monash University.^23^ Any discrepancy in the data collected was resolved via discussion or adjudication by the senior reviewer (MJP). Before data collection, a pilot test of the data collection form was performed on a random sample of 10 systematic reviews and the form was adjusted as necessary. The full data collection form (supplemental data) includes a subset of items used in previous evaluations of completeness of reporting^9^ ^10^ along with additional items to capture some issues not previously examined. The wording of items in the data collection form was matched to previous evaluations^9^ ^10^ to facilitate comparison.

The form consisted of three sections (table 1). The first section captured general characteristics of the review, which were all extracted manually except for the country of the corresponding author, which was extracted using R code adapted from the easyPubMed package version 2.13.^24^ ^25^ The interventions were classified as health, social, behavioural, or educational interventions (see definitions in the supplemental data). The second section consisted of items describing the review’s reporting characteristics, the index meta-analysis (defined as the first meta-analysis mentioned in the abstract/results sections), and its data sharing characteristics. All of the reporting items evaluated are recommended in the 2009 PRISMA statement (in either the main checklist or the explanation and elaboration document^26^), except for the items on whether search strategies for all bibliographic databases and non-database sources were reported. To facilitate our analysis of the impact of reporting guidelines, we also recorded whether the authors self-reported using a reporting guideline, defined as any document specifying essential items to report in a systematic review (eg, PRISMA, MECIR (Methodological Expectations of Cochrane Intervention Reviews), MECCIR (Methodological Expectations of Campbell Collaboration Intervention Reviews) standards).

**Table 1:** Items for data collection and data sources (see the supplemental data (S4 appendix) for further details)

Source and section	Data items
Systematic review*	
General characteristics of the systematic review	Title; journal name; corresponding author’s country; source of funding for the review; conflicts of interest of review authors; number of studies included in the review; types of participants and interventions investigated.
Reporting characteristics of the systematic review	Whether a reporting guideline was cited; whether a protocol or registration record for the review was cited; whether eligibility criteria for participants, interventions, comparators, outcomes, and study designs were reported; what details of the search methods were reported (including which databases, the interface used to search them, the years of coverage, date of the search, and whether a full Boolean search logic—using operators such as “AND,” “OR,” “NOT”—was reported); what method of study selection, data collection, and risk-of-bias assessment authors reported using; what software (and packages) authors reported using; whether the number of records yielded by the searches were reported overall and for each database; whether any full text articles excluded from the review were cited.
Reporting characteristics of the index (first reported) meta-analysis	Outcome domain investigated; number of included studies; effect measure used; whether methods required to prepare data for meta-analysis were reported; whether the meta-analysis model used was reported; whether the meta-analysis method used was reported; whether the between-study (heterogeneity) variance estimator used was reported; whether summary statistics for each included study were reported; whether effect estimates and measures of precision for each included study were reported.
Sharing characteristics of the systematic review	Whether a data or code availability statement appeared in the review; which types of files were made publicly accessible either as a supplementary file or uploaded to a repository (eg, files containing data used in all analyses, analytical code used to generate results, files containing citations of all records that were screened and excluded); whether files shared had a persistent identifier (eg, doi) or license (eg, cc-by) applied to them.
Journal website	Journal name; whether the journal only publishes evidence syntheses (ie, systematic reviews and their protocols); whether the journal has a data or code sharing policy, or both; whether sharing data and/or issuing a data availability statement is mandatory for systematic reviews published by the journal.

* Includes the main report and any supplementary file(s), and the review protocol (if the authors specified that the relevant information was included).

The final section captured the data sharing policy of the journal where the article was published. A data sharing policy refers to the journal’s requirements and expectations regarding public sharing of data and code used in the review. Web archives (https://web.archive.org/) were used to retrieve the version of the policy published before 1 November 2020.

We collected data from the main report of the systematic review, any supplementary file provided on the journal server or any cited repository, the review protocol (if the authors specified that the relevant information was contained therein), and journal websites (table 1). In the event of discrepancies between the protocol and the main report, we gave preference to data from the main report.

### Secondary use of data collected on systematic reviews from 2014

We obtained the dataset previously collated by Page et al,^10^ which included data on completeness of reporting and sharing of review data in a random sample of 110 systematic reviews of health interventions indexed in Medline in February 2014. The reviews included in the 2014 dataset were drawn from a random sample of 300 systematic reviews of health research that answered questions of intervention efficacy, diagnostic test accuracy, epidemiology, or prognosis, 110 of which evaluated the effect of health interventions and met the same eligibility criteria that the 2020 reviews had to meet (apart from year of publication). We extracted individual review data from the 2014 dataset for all reporting and sharing items that were worded the same or similarly as the items collected in the 2020 sample. Where necessary, we recoded data in the 2014 sample to ensure harmonisation with the 2020 sample. We did not collect any additional data on the systematic reviews (or the journals they were published in) in the 2014 sample. Given the systematic reviews in 2014 were identified via Medline only, whereas the systematic reviews in 2020 were identified via five databases (PubMed, Science Citation Index, Social Sciences Citation Index, Scopus, and Education Collection), we determined how many of the included reviews from 2020 happened also to be indexed in Medline, to ensure the comparison between years was appropriate.

### Data analysis

We summarised general and reporting characteristics of the included systematic reviews using descriptive statistics (eg, frequency and percentage for categorical items, median and interquartile range for continuous items). We calculated risk ratios to quantify differences in the percentage of reviews meeting indicators of “completeness of reporting” and “sharing of review materials” between the following groups:

- Reviews published in 2020 in an evidence synthesis journal (defined as a journal which has a strong or exclusive focus on systematic reviews and their protocols, as identified from the journal website’s aims and scope sections) versus reviews published elsewhere
- Reviews of health interventions published in 2020 versus reviews of health interventions published in 2014
- Reviews published in 2020 reporting use of a reporting guideline (eg, PRISMA) versus reviews published the same year not reporting such use
- Reviews published in 2020 in journals with a data sharing policy versus journals without one
- Reviews published in 2020 in journals with a policy that mandates either data sharing or declaration of data availability, irrespective of whether the policy applies universally to all studies or specifically to systematic reviews, versus journals without such a policy.

Risk ratios and Wald type normal 95% confidence intervals were calculated using the epitool package version 0.5-10.1 (R version 4.0.3).^27^ Where the numerators were small (<5) in either group, or the outcome was very rare (<5%) in either group, we instead used penalised likelihood logistic regression (implemented via the logistf package version 1.24 in R).^28^ Penalised likelihood logistic regression has been shown to improve estimation of the odds ratio and its confidence interval for rare events or unbalanced samples.^29^ ^30^ The odds ratios from these models can be interpreted as risk ratios when the events are rare in both groups.^31^ The risk ratios and their 95% confidence intervals were displayed using forest plots (implemented via the forestplot package version 1.10.1 in R).^32^ Rather than relying on statistical significance when interpreting risk ratio associations (ie, claiming that an association exists when the 95% confidence interval did not include the null), we defined an equivalence range for all comparisons as 0.9-1.1. Any risk ratio less than 0.9 or greater than 1.1 (ie, a 10% difference in rate of reporting in either direction) was deemed to be an important difference. Since no previous study has identified a meaningful threshold for important changes in reporting in systematic reviews, this equivalence range was determined based on consensus between investigators. Assuming an item was reported by 50% of reviews in 2014, a risk ratio of 1.1 reflects that the item was reported by 55% of reviews in 2020 (a difference of five percentage points). If the reporting rate in 2014 is higher than 50% (eg, 80%), the threshold to be considered an important difference will be higher (ie, eight percentage points).

We conducted two post hocsensitivity analyses. The first was conducted by excluding Cochrane reviews because they are subjected to strict editorial processes to ensure adherence to methodological conduct and reporting standards, and the second by excluding reviews on covid-19 owing to concerns about short publication turnarounds, which could have an impact on reporting quality.^33^

### Patient and public involvement

We did not involve patients or members of the public directly when we designed our study, interpreted the results, or wrote the manuscript, because our focus was to identify problems in how researchers report their work in scientific journals with a predominantly scientific readership. However, the idea for our study arose from our concerns as people who interact with the healthcare system that incomplete reporting can lead to undue trust being placed in the findings of flawed systematic reviews, potentially leading to ineffective or harmful treatments being delivered. We asked a member of the public to read our manuscript after submission to ensure it was understandable to the general reader.

## Results

### Results of the search

**Fig 1:**
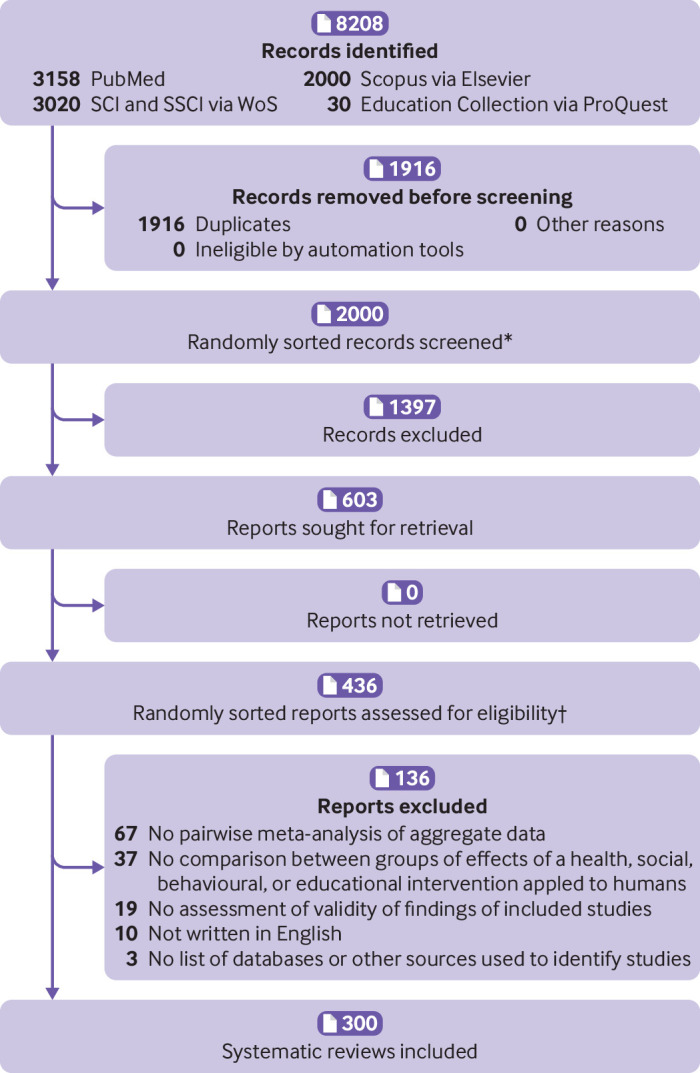
PRISMA 2020 flow diagram of identification, screening, and inclusion of systematic reviews. *6292 unique records remained after duplicates were removed, but only the first 2000 randomly sorted records were needed to screen in order to reach the required target sample size. †Only the first 436 of 603 full text reports retrieved were needed to screen in order to reach the required target sample size

Our search retrieved 8208 records (fig 1). Of the first 2000 randomly sorted titles and abstracts that were screened, we considered 603 as potentially eligible and retrieved the full text for screening. We only needed to screen the first 436 randomly sorted full text reports to reach our target sample size of 300. Citations of all records identified, screened, excluded, and included are available on the Open Science Framework (doi:10.17605/OSF.IO/JSP9T).

### General characteristics of systematic reviews

Among the 2020 sample (n=300), half of the systematic reviews (n=151, 50%) had a corresponding author based in one of three countries: China (n=96, 32%), the US (n=31, 10%), and the UK (n=24, 8%) (table 2). The reviews included a median of 12 studies (interquartile range 8-21), with index meta-analyses including a median of six studies (interquartile range 4-10). Most reviews (n=215, 72%) included a financial disclosure statement, of which 97 (32%) declared no funding. Most corresponding authors (n=251, 84%) declared having no conflict of interest. Common software used for meta-analysis were Review Manager (n=189, 63%), Stata (n=73, 24%), and R (n=33, 11%).

**Table 2:** Descriptive characteristics of systematic reviews indexed in 2020

Item	Frequency (%)
**General**	
Median No (IQR) of databases searched	4 (3-5)
Median No (IQR) of studies included in review	12 (8-21)
Median No (IQR) of studies in index meta-analysis	6 (4-10)
Country of corresponding author	
China	96/300 (32)
USA	31/300 (10)
UK	24/300 (8)
Other	149/300 (50)
Source of funding	
Non-profit	112/300 (37)
For profit	3/300 (1)
Both for profit and non-profit	3/300 (1)
No funding	97/300 (32)
Not reported	85/300 (28)
Conflict of interest	
Present	30/300 (10)
Not present	251/300 (84)
Not declared	19/300 (6)
ICD-11 category investigated	
Endocrine, nutritional, or metabolic diseases	36/300 (12)
Diseases of the digestive system	36/300 (12)
Diseases of the musculoskeletal system or connective tissue	35/300 (12)
Diseases of the circulatory system	30/300 (10)
Other	163/300 (54)
Type(s) of intervention	
Drug	102/300 (34)
Non-drug	189/300 (63)
Both	9/300 (3)
Area(s) of intervention	
Health	294/300 (98)
Behavioural	28/300 (9)
Educational	4/300 (1)
Social	5/300 (2)
Citing a reporting guideline	245/300 (82)
**Reporting of the review process**	
Protocol/registration record cited	
Both protocol and registration record cited	1/300 (<0.5)
Only a protocol cited	13/300 (4)
Only a registration record cited	112/300 (37)
Neither	174/300 (58)
Eligibility criteria stated	
Participants	275/300 (92)
Interventions/exposures	296/300 (99)
Comparators	267/300 (89)
Outcomes	298/300 (99)
Study design	278/300 (93)
Type(s) of eligible study design	
Randomised studies	168/278 (60)
Non-randomised studies	20/278 (7)
Both	90/278 (32)
Search interface reported (eg, Ovid for Medline)	
For all databases	76/300 (25)
For some databases	36/300 (12)
Not reported	188/300 (63)
Dates of coverage of databases reported	
Both exact start and end dates	136/300 (45)
Both start and end dates but not as exact dates (eg, from inception to May 2020)	105/300 (35)
Only start or end date	50/300 (17)
Not reported	9/300 (3)
Exact last date of search reported*	72/300 (24)
Search strategy for databases reported	
Boolean logic for all databases	81/300 (27)
Boolean logic for some databases	133/300 (44)
List of MeSH and free text terms only	12/300 (4)
List of MeSH terms only	8/300 (3)
List of free text terms only	59/300 (20)
Not reported	7/300 (2)
Trials register searched	64/300 (21)
Other electronic sources searched	102/300 (34)
Search strategy for trial register/other sources reported	24/140 (17)
Method of screening	
All studies screened by at least two authors	196/300 (65)
At least two authors were involved in either titles/abstracts or full text screening, but unclear for the other step	22/300 (7)
Different methods were applied for titles/abstracts and full text screening	7/300 (2)
All studies screened by one author and a subset by another author	4/300 (1)
All studies screened by one author with the use of an automation tool	0/300 (0)
All studies screened by one author only	4/300 (1)
Not reported	67/300 (22)
Method of data collection	
All data collected by two authors	208/300 (69)
All data collected by one author with verification by another	16/300 (5)
All data collected by one author only	4/300 (1)
Other arrangements	1/300 (<0.5)
Not reported	71/300 (24)
Method of risk-of-bias assessment	
All studies assessed by two authors	173/300 (58)
All studies assessed by one author with verification by another	7/300 (2)
All studies assessed by one author only	5/300 (2)
Not reported	115/300 (38)
Risk-of-bias assessment reported for each study	231/300 (77)
Total records retrieved reported	300/300 (100)
Records retrieved per database reported	126/300 (42)
Software used for meta-analysis	
Review Manager	189/300 (63)
Stata	73/300 (24)
R	33/300 (11)
Comprehensive Meta-Analysis	27/300 (9)
SPSS	4/300 (1)
SAS	1/300 (<0.5)
Other	13/300 (4)
Not reported	3/300 (1)
Software details reported	
Both analysis package and software version reported	28/297 (9)
Only analysis package reported	8/297 (3)
Only software version reported	242/297 (81)
Neither	19/297 (6)
At least one excluded article cited	65/300 (22)
**Index meta-analysis**	
Measure of effect used	
Risk ratio	72/300 (24)
Odds ratio	71/300 (24)
Hazard ratio	13/300 (4)
Risk difference	3/300 (1)
Mean difference	76/300 (25)
Standardised mean difference	63/300 (21)
Other	2/300 (1)
Method of data preparation reported	101/300 (34)
Meta-analysis model reported (eg, fixed effect, random effects)	294/300 (98)
Meta-analysis method reported (eg, Mantel-Haenszel, inverse variance)	218/300 (73)
Heterogeneity variance estimator reported (eg, DerSimonian-Laird)	50/235 (21)
Summary statistics reported for each study	215/300 (72)
Effect estimate and measure of precision reported for each study	288/300 (96)
**Sharing of data and materials used in analyses**	
Data/code availability statement present	93/300 (31)
Types of data shared	
Unprocessed extracted data	9/300 (3)
Data conversions performed	1/300 (<0.5)
Data used in analyses	12/300 (4)
Analytical code	2/300 (1)
Citations of all screened studies	2/300 (1)
Metadata of shared files	1/300 (<0.5)
Any of the above	20/300 (7)
Method(s) of sharing	
Supplementary files	20/20 (100)
Open access repository	1/20 (5)
Institutional repository	1/20 (5)
DOI cited for shared data	2/2 (100)
License stated for shared data	2/2 (100)
**Journal that publishes the review**	
Specialised in evidence syntheses (eg, Cochrane Database of Systematic Reviews)	14/300 (5)
Data policy stated in guideline for authors	199/300 (66)
Sharing of data and/or analytical code is encouraged, but it is not mandatory for publication in the journal, and a data availability statement is not required.	112/199 (56)
Sharing data and/or analytical code is not mandatory for publication of systematic reviews, but a data availability statement, which contains links to shared data or reasons for not sharing data, must be provided	51/199 (26)
Sharing data and/or analytical code is a condition of publication of systematic reviews by the journal	36/199 (18)

ICD-11=international classification of diseases, 11th revision; IQR=interquartile range.

* We recorded whether the author specified the date of the last search, which, in practice, might not be the same as the end date of the search range.

The included reviews covered a wide range of topics. The intervention was classified as a health intervention in nearly all reviews (n=294, 98%), and as a social, behavioural, or educational intervention in 37 (12%) (some reviews examined both types of interventions). Almost two thirds of the reviews (n=198, 66%) examined the effects of non-drug interventions. Of 24 ICD-11 (international classification of diseases, 11th revision) categories of diseases and conditions, our sample of reviews captured 23 categories. The top four categories (endocrine, nutritional, or metabolic diseases, diseases of the digestive system, diseases of the musculoskeletal system, and diseases of the circulatory system) accounted for 46% (n=137) of all systematic reviews.

The included systematic reviews were published across 223 journals. Five journals (accounting for 5% of all systematic reviews) specialised in evidence synthesis; 140 journals (accounting for 66% of all systematic reviews) outlined a data sharing policy in the instruction page for authors (supplemental data).

The general characteristics of the 2014 sample (n=110) have been described elsewhere.^10^ In brief, the 2014 sample was similar to the 2020 sample in many aspects, such as the sample size of each review (median 13 studies, interquartile range 7-23), size of the index meta-analysis (median 6 studies, interquartile range 3-11), and the prevalence of non-drug reviews (n=55, 50%). Like the 2020 sample, the reviews in 2014 were published in a wide range of journals (n=63), addressed several clinical topics (19 ICD-10 categories), and predominantly had corresponding authors from China, the UK, and Canada (n=55 combined, 50%).

### Completeness of reporting of reviews in systematic reviews from 2020

Of the items we examined, the most frequently reported included the total number of records yielded from searches (n=300, 100%), a declaration of review authors’ conflicts of interest (or lack of) (n=281, 94%), each of the PICOS (participants, interventions, comparators, outcomes, and study designs) components of the eligibility criteria (n=267-298, 89-99%), the meta-analysis model (eg, fixed effect) used (n=294, 98%), and the effect estimates, together with the measures of precision, for each study included in the index meta-analysis (n=288, 96%) (table 2). On the other hand, several items were reported in 50-80% of reviews. These items included the funding source for the review (n=215, 72%), start and end dates of coverage of databases searched (n=241, 80%), a full boolean search logic for at least one database (n=214, 71%), methods used to screen studies (n=233, 78%), methods used to collect data (n=229, 76%), methods used to assess risk of bias (n=185, 62%), the meta-analysis method (eg, Mantel-Haenszel, inverse variance) used (n=218, 73%), and summary statistics for each study included in the index meta-analysis (n=215, 72%).

Several items were reported in fewer than 50% of reviews. These items included a registration record (n=113, 38%) or protocol (n=14, 5%) for the review, the interfaces used to search databases (eg, Ovid, EBSCOhost) (n=112, 37%), a search strategy for sources that are not bibliographic databases (n=24 of 140 reviews that indicated they searched other sources, 17%), the number of records retrieved for each database (n=126, 42%), a citation for at least one excluded article (n=65, 22%), methods of data preparation (eg, data conversion, calculation of missing statistics) (n=101, 34%), and the heterogeneity variance estimator used for the index meta-analysis (n=50 of 235 reviews that performed a random effects meta-analysis, 21%).

### Sharing of data, analytical code, and other review materials in systematic reviews from 2020

In our 2020 sample, 20 systematic reviews (7%) made data files or analytical code underlying the meta-analysis publicly available, which included two reviews (1%) that shared analytical code. All of these reviews shared these data via supplementary files; two reviews additionally hosted data and analytical code in a public repository. The most commonly shared materials were data files used in analyses, such as RevMan files (n=12, 4%).

### Changing patterns of reporting between 2014 and 2020

**Fig 2:**
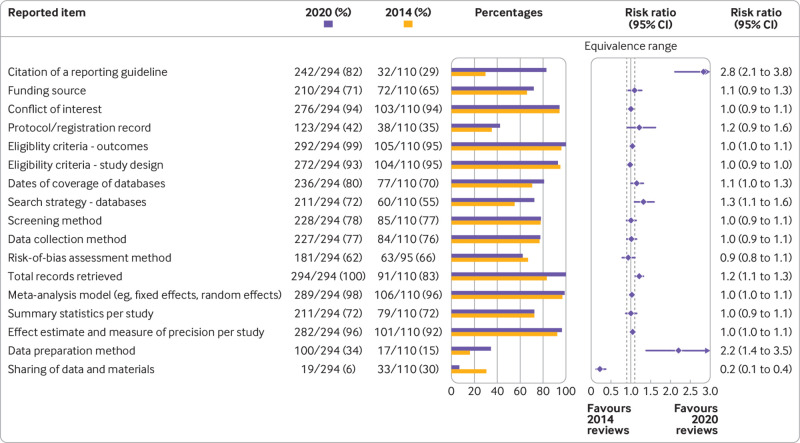
Frequency of reporting items between systematic reviews indexed in 2014 and 2020. Equivalence range=0.9-1.1

Of the 300 systematic reviews from 2020, 294 were systematic reviews of health interventions, which we compared with 110 reviews of health interventions from 2014. We determined that 87% of the 294 reviews from 2020 were indexed in Medline; given this high percentage, we consider the comparison with systematic reviews indexed in Medline in 2014 to be appropriate. Compared with the 2014 reviews, systematic reviews indexed in 2020 cited a reporting guideline more frequently (82% *v* 29%) and were more likely to report a full search strategy for at least one database (72% *v* 55%), the total number of records retrieved (100% *v* 83%), and data preparation methods (34% *v* 15%); 95% confidence intervals for all risk ratios exceeded the upper limit of the equivalence range (fig 2). For five reporting items, frequencies in both years were similarly high (>90%), leaving little room for improvement. For six other reporting items, frequency of reporting in both years was less than 80% and the estimated differences between years were uncertain because the 95% confidence intervals included the equivalence range (fig 2). In a sensitivity analysis excluding Cochrane reviews from both samples (supplemental data), some existing differences became more pronounced, or 95% confidence intervals narrowed.

### Impact of reporting guidelines, journal type, and data sharing policies on reporting in systematic reviews from 2020

**Fig 3:**
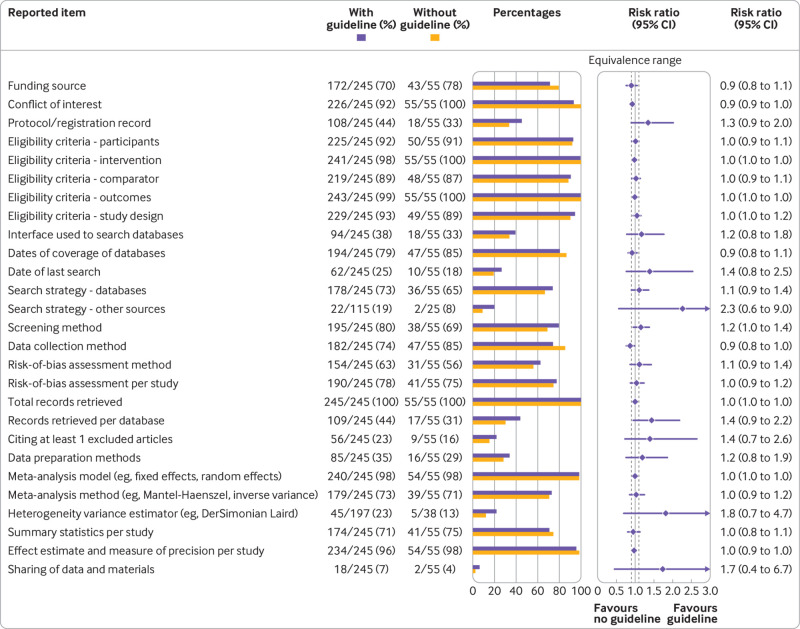
Association between citation of a reporting guideline and reported items. Equivalence range=0.9-1.1

Of the 300 reviews from 2020, 245 (82%) reported using a reporting guideline. No evidence indicated that such reviews were more completely reported than reviews not using a guideline, because for all reporting items, 95% confidence intervals for the risk ratios crossed the equivalence range (fig 3). However, of the 27 reporting items compared, seven were reported at a high frequency (>90%) in both groups, leaving little scope for a disparity. We conducted a sensitivity analysis by excluding systematic reviews on covid-19 (n=6) from both groups, but no notable changes were observed (supplemental data).

**Fig 4:**
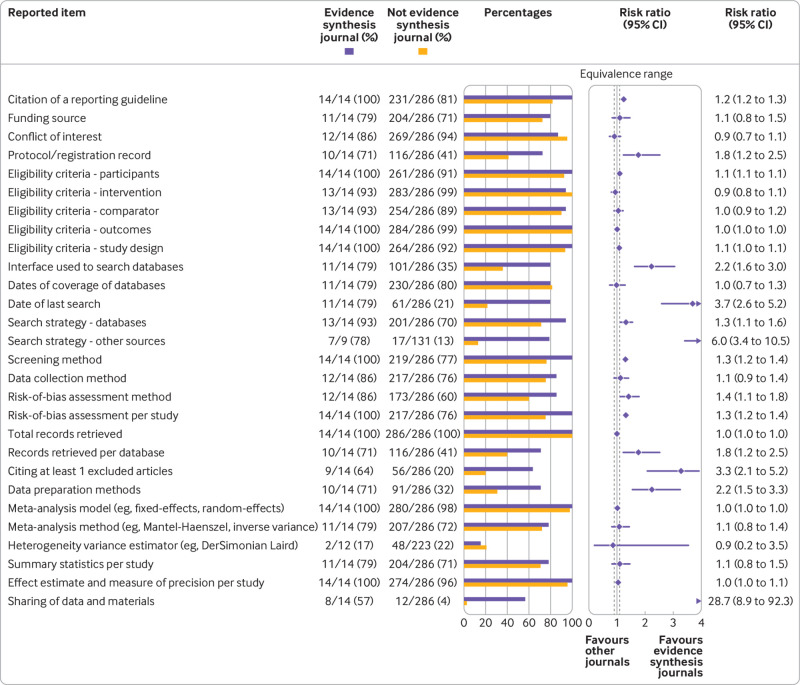
Association between journal type and reported items. Equivalence range=0.9-1.1

Only 14 systematic reviews from 2020 were published in specialist evidence synthesis journals, including eight Cochrane reviews. Such reviews were reported more completely than reviews published elsewhere, with 95% confidence intervals for risk ratios exceeding the upper limit of the equivalence range for 14 of 28 reporting items compared (fig 4). Such items included those that have received limited attention in previous meta-research studies, such as the interface used to search bibliographic databases (79% *v* 35%), a search strategy for non-database sources (78% *v* 13%), citation for at least one excluded study (64% *v* 20%), and availability of data and materials (57% *v* 4%).

**Fig 5:**
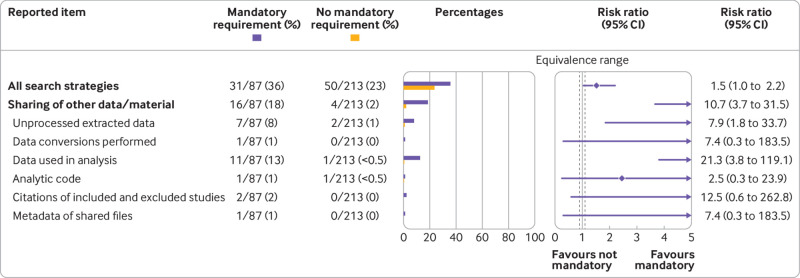
Association between journals’ data sharing requirements and reported items. Mandatory requirement=a mandatory instruction for sharing of data and materials, or in the absence of such data, a data availability statement stating why data were not shared and whether data are available on request. Equivalence range=0.9-1.1

Systematic reviews from 2020 published in a journal with a mandatory requirement for data sharing or declaration of data availability were more likely than reviews published elsewhere to share any data or materials (18% *v* 2%) (fig 5). Similar findings were observed when comparing between reviews published in journals with any data sharing policy (mandatory or otherwise) and journals without one (supplemental data).

## Discussion

Findings from our examination of 300 randomly selected systematic reviews indexed in 2020 indicate suboptimal reporting of several items, such as the reporting of a review protocol (5%) or registration entry (38%), search strategy for all databases (27%), methods of data preparation (eg, imputing missing data, data conversions) (34%), and funding source for the review (72%). Other meta-research studies reported similar frequencies of reporting of review protocols (17%),^6^ preregistration records (22%),^6^ full search strategies for all databases (14%),^7^ handling of missing data (25%),^4^ and the funding source for the review (62%).^6^ Some discrepancies in these results can be attributed to differences in assessment criteria and the disciplines studied.^34^ In our sample of reviews indexed in 2020, citation of reporting guidelines was common (82%), but no evidence was found indicating that reviews that cited a guideline were reported more completely than reviews that did not, an observation shared by Wayant et al.^4^ We also reported a scarcity of the sharing of data and code files (7%), which is within the range of previously reported results (0.6-11%).^4^ ^8^ ^35^ Journals’ open data policies were found to have positive impacts on the frequency of sharing certain types of review data and analytical code, which aligns with evaluations of other study designs.^36^ ^37^

### Strengths and limitations of the study

Although this topic has been explored in other meta-research studies,^2^ ^3^ ^4^ ^5^ ^6^ ^7^ ^8^ our study offers several methodological advantages. Firstly, our assessment of reporting captured several recommended reporting items in the PRISMA 2020 statement^38^ which have not previously been explored. Secondly, most previous meta-research studies on this topic used the 2009 PRISMA checklist to evaluate reporting,^15^ in which several reporting items comprise multiple elements (eg, item 10 reads, “Describe method of data extraction from reports (such as piloted forms, independently, in duplicate) and any processes for obtaining and confirming data from investigators).” Simply recording “reported” for such an item does not clearly distinguish which elements in the item were actually reported. By contrast, the criteria we used to evaluate systematic reviews allowed for a more comprehensive and granular assessment of reporting in systematic reviews. Thirdly, our sample consists of systematic reviews published a few months before the PRISMA 2020 statement was released, and thus provides a useful benchmark for future meta-research studies to explore whether changes in reporting occurred after the release of PRISMA 2020. Fourthly, we searched several databases to identify eligible systematic reviews, and our sample was not limited to a specific topic or journal. Fifthly, our study captured not only the frequency of data sharing, but also the types of systematic review data, code, and materials being shared. Lastly, we compared our 2020 sample with a 2014 sample that was retrieved and evaluated using the same criteria,^9^ ^10^ thus minimising the impact of methodological variations.

Nonetheless, our study was not without limitations. We used web archives to determine the journal’s policies on data sharing before 1 November 2020 (ie, just before the reviews in our sample were indexed in databases), but it was impossible to confirm with certainty the journal data policy that reviewers would have seen at the time they submitted their systematic review. As a cross sectional study, our results should be viewed as generating hypotheses rather than proving a causal association. Some items were reported by fewer than 50 reviews, which caused uncertainty in interpreting their risk ratios. Despite intending to include systematic reviews of the effects of health, social, behavioural, and educational interventions, the vast majority of reviews evaluated the effects of a health intervention. Therefore, our findings are less generalisable to systematic reviews of the other types of interventions. Lastly, our findings do not necessarily generalise to systematic reviews indexed in databases other than the ones we searched, or to systematic reviews written in languages other than English.

### On reporting of systematic reviews

We observed few notable improvements in reporting between 2014 and 2020 for several possible reasons. Firstly, several items were already reported frequently in 2014 (eg, reporting of competing interests, eligibility criteria, meta-analysis models, effect estimate for each study), leaving little opportunity for improvement. Secondly, some reporting items we examined have only been recommended for reporting recently (eg, in the PRISMA 2020 statement published in March 2021),^38^ such as the search strategy for all databases or the availability of data or analytical code. As such, authors of reviews in our study using older reporting guidelines might not have felt compelled to report these details in either 2014 or 2020.

Most systematic reviews in 2020 cited a reporting guideline, yet such guideline use was not clearly associated with improved reporting for any of the assessed items. This uncertain association between citation of a reporting guideline and completeness of reporting challenges the assumption that referencing a reporting guideline guarantees adherence to the guideline. In reality, other factors could have affected the authors’ decision not to report certain items. Firstly, authors might assume that reporting of methods used for one process implies that the same approach was used for another process. For example, we observed among our sample a tendency to report the reviewer arrangement only for the screening stage, and not for the subsequent data collection or risk-of-bias assessment stages. Secondly, authors might incorrectly assume that the meta-analysis methods can always be deduced from the packages and software used, or by reading the forest plot. Such inference of methods is not always possible,^39^ as different meta-analysis software have different options and default settings.^40^ Thirdly, some items are difficult to report if the reviewer had not recorded relevant details during the conduct of the review (eg, number of records excluded, data conversions performed). Fourthly, nearly all of the items reported in less than 50% of reviews, such as the interface used to search databases and meta-analysis method used, are recommended only in the explanation and elaboration document of the 2009 PRISMA statement, so these important elements might have been missed by authors using only the PRISMA checklist to guide reporting. In future, we recommend interviews be conducted with review authors to explore their understanding of reporting guidelines and identify challenges in reporting of reviews. Furthermore, interventions should be developed and evaluated to help improve reporting (such as a computer based tool to break down the PRISMA reporting recommendations—both those appearing in the main checklist and those in the explanation and elaboration document—into digestible steps for first time reviewers^41^ ^42^) and aid peer reviewers’ ability to detect incomplete reporting.

### On data sharing in systematic reviews

The low rate of data and code sharing can be attributed to several factors. Firstly, the issue of data sharing for systematic reviews has received relatively little attention until recently. A recommendation to report whether data, code, and other materials are publicly available was only recommended in the PRISMA 2020 statement (published in March 2021), while our sample of systematic reviews was published before December 2020. Secondly, there has been a rise in percentage of non-Cochrane reviews between 2014 and 2020. Unlike Cochrane reviews, which are routinely published together with RevMan files containing meta-analysis data, non-Cochrane reviews are not always subjected to data sharing requirements. Thirdly, some motivational, educational, and technical barriers to data sharing cannot be sufficiently dealt with by data sharing policies, such as lack of technical expertise and time, lack of data management templates to facilitate sharing of review data, concerns about data ownership, fear of criticism, and lack of career incentives.^43^ ^44^ Some studies have explored these barriers in general academia, but we are uncertain whether researchers in evidence synthesis face all of these barriers, face only some of them, or face unidentified barriers unique to systematic reviews and meta-analyses. Future studies in the REPRISE project will explore systematic reviewers’ perspectives to answer these questions.^20^

Lastly, our findings also highlight the important role of supplementary files or public repositories for data sharing in systematic reviews. Web based supplementary files and public repositories enable authors to share data and materials necessary to validate the review process while keeping the main article concise and relevant to lay readers.^10^ For example, authors can outline in a separate file all search strategies specific to databases (eg, Saeteaw et al^45^), excluded studies at each stage of screening (eg, Bidjan et al^46^), and complete data for all meta-analyses (eg, Hill et al^47^). Data sharing via supplementary files or public repositories is an effective tool to improve reproducibility of systematic reviews and should be made a standard practice. Concerted efforts around data infrastructure, fair use guidelines, and a supportive environment are required to make data sharing a standard practice.^48^ ^49^ ^50^

### Conclusion

Incomplete reporting of several recommended items in systematic reviews persists, even in reviews that claim to have followed a reporting guideline. Data sharing policies could be an effective strategy to promote sharing of systematic review data and materials.

**What is already known on this topic**

Complete reporting of methods and results, as well as sharing data and analytical code, enhances transparency and reproducibility of systematic reviews; the extent of complete reporting and sharing of data or analytical code among systematic reviews needs to be comprehensively assessed
Use of reporting guidelines, which are designed to improve reporting in systematic reviews, is increasing; it is unclear whether this increase has affected reporting of methods and results in systematic reviews
More journals are adopting open data policies which aim to promote data sharing; the impact of these policies on the sharing of data and analytical code in systematic reviews is also unclear

**What this study adds**

Incomplete reporting of several recommended items in systematic reviews persists; sharing of review data and analytical code is currently uncommon (7%)
An increase in self-reported use of a reporting guideline was observed between 2014-2020; however, there was no evidence that reviews using a reporting guideline were more completely reported than reviews not using a guideline
Reviews published in 2020 in journals that mandated either data sharing or inclusion of data availability statements were more likely to share their review materials (eg, data, code files)

## Data Availability

All datasets and analytical code can be found on the Open Science Framework (https://osf.io/jsp9t/; doi:10.17605/OSF.IO/JSP9T).

## Web extra

Extra material supplied by authors

Web appendix: Supplemental data

## Notes

Contributors: P-YN curated the data, performed the formal analysis, contributed to the investigation and methodology, and wrote and prepared the original draft. RK, ZA, and SM contributed to the investigation and the writing and editing of the manuscript. JEM conceptualised, supervised, and validated the study, and contributed to the writing and editing of the manuscript. MJP acquired the funding, conceptualised and supervised the study, and contributed to methodology and the writing and editing of the manuscript. SEB, NRH, DGH, SK, DM, SN, DN, PT, and VAW interpreted the results and contributed to the writing and editing of the manuscript. All authors contributed to writing the manuscript, approved the final version, and had final responsibility for the decision to submit for publication. MJP is guarantor. The corresponding author attests that all listed authors meet authorship criteria and that no others meeting the criteria have been omitted.

The lead authors (P-YN and MJP) affirm that the manuscript is an honest, accurate, and transparent account of the study being reported; that no important aspects of the study have been omitted; and that any discrepancies from the study as originally planned (and, if relevant, registered) have been explained.

Dissemination to participants and related patient and public communities: We plan to disseminate results of this study at national and international conferences, via seminars and workshops teaching systematic review methods (targeting clinicians, guideline developers, researchers, and other stakeholders) and via Twitter.

Provenance and peer review: Not commissioned; externally peer reviewed.
