# Supplementary material for: Changing patterns in reporting and sharing of review data in systematic reviews with meta-analysis of the effects of interventions: cross sectional meta-research study
Source: BMJ. 2022 Nov 22;379:e072428. doi: 10.1136/bmj-2022-072428 (PMC9679891; doi:10.1136/bmj-2022-072428)

Supplemental Data

TITLE PAGE

Appendix S1. Deviations from the REPRISE project protocol..2

Appendix S2. Eligibility criteria for study inclusion..3

Appendix S3. Search strategy 4

Appendix S4. Data extraction form 5

Appendix S5. List of journals and their policies 15

Appendix S6: Comparison between reviews of health interventions in 2014 vs 2020 21

Figure S1: Sensitivity analysis – Frequency of reporting items between systematic reviews published in 2014 and 2020 23

Figure S2: Sensitivity analysis – Relationship between citation of reporting guidelines and reported items 24

Figure S3: Relationship between journal's presence of a data sharing policy and reported items..26

Appendix S1. Deviations from the REPRISE project protocol

**Original plan**	**Revised plan**	**Reason for modification**
We will use risk ratios with 95% confidence intervals to examine differences in percentages of each indicator between reviews in different categories (e.g. 2020 versus 2014 reviews).	When the numerators were small (<5) in either group, or the outcome was very rare (<5%) in either group, rather than using risk ratios, we instead used penalised likelihood logistic regression and generated odds ratios.	Penalised likelihood logistic regression has been shown to improve estimation of the risk ratio and its confidence interval for rare events or unbalanced samples. The odds ratios from these models can be interpreted as risk ratios when the events are rare in both groups.
We will use risk ratios with 95% confidence intervals to examine differences in percentages of each indicator between reviews published in journals with versus without a mandatory data or code sharing policy	We will use risk ratios with 95% confidence intervals to examine differences in percentages of each indicator between reviews published in journals with versus without a policy that mandates either data sharing or declaration of data availability.	Too few journals had a policy that mandated data sharing, so we expanded this category to those journals which mandated inclusion of a data availability statement.
We did not plan to define an equivalence range to interpret risk ratio associations.	We defined an equivalence range for all comparisons as 0.9 to 1.1 – any risk ratio less than 0.9 or more than 1.1 was deemed as an important difference.	We set an equivalence range in this paper because there are so many statistical tests being undertaken. With all these tests, there is a temptation to focus on those that are statistically significant, even if unimportant.
We did not plan to conduct any sensitivity analyses.	We conducted two post-hoc sensitivity analyses, the first by excluding Cochrane reviews, and the second by excluding reviews on COVID-19.	We excluded Cochrane reviews because they are subjected to strict editorial processes to ensure adherence to methodological conduct and reporting standards (unlike systematic reviews published in other journals). We excluded reviews focusing on COVID-19 due to concerns about short publication turnarounds, which could have an impact on reporting quality.

Appendix S2. Eligibility criteria for study inclusion

**Title and abstract screening**
1. Based on the title/abstract, the article appears to be a completed systematic review with meta-analysis of studies (i.e. not a protocol for a systematic review, nor a systematic review without meta-analysis, nor an overview of systematic reviews, nor a scoping review); and
2. The article appears to have compared the effects of at least two health, social, behavioural or educational interventions applied to humans. That is, any intervention designed to improve health ("a state of complete physical, mental and social well-being and not merely the absence of disease or infirmity"), promote social welfare and justice, change behaviour, or improve educational outcomes.
**Full-text screening**
1. The article was written in English; and
2. The review objective or question was clearly stated in the Introduction or Methods section; and
3. The source(s) used to search for studies were reported; and
4. Methods used to assess validity of the findings of the included studies (e.g. risk of bias assessment, methodological quality assessment) were reported in the Methods or Results section; and
5. The review included randomised or non-randomised studies (or both) comparing the effects of at least two health, social, behavioural or educational interventions designed to improve health, promote social welfare and justice, change behaviour, or improve educational outcomes in humans. That is, any intervention designed to improve health ("a state of complete physical, mental and social well-being and not merely the absence of disease or infirmity"), promote social welfare and justice, change behaviour, or improve educational outcomes.
6. References to all studies included in the review were cited, either in the standard reference list or in a supplementary file (full bibliographic citations of included studies must have been presented for the systematic review to be considered eligible).
7. The review presented at least one pairwise meta-analysis of aggregate data, including at least two studies, using any effect measure (e.g. mean difference, risk ratio). Network meta-analyses were eligible if they included at least one direct (i.e. pairwise) comparison that fulfilled the abovementioned criterion. Meta-analyses of individual participant data were excluded.

Appendix S3. Search strategy

All searches were run on December 3rd, 2020.

PubMed

(meta-analysis[PT] OR meta-analysis[TI] OR systematic[sb]) AND 2020/11/02:2020/12/02[EDAT]

Science Citation Index Expanded (SCI-EXPANDED) and Social Sciences Citation Index (SSCI) via Web of Science

(TI=meta-analysis OR AB=meta-analysis OR TS=meta-analysis OR TI="systematic review" OR AB="systematic review" OR TS="systematic review") AND LANGUAGE: (English) AND DOCUMENT TYPES: (Article OR Review)

Indexes=SCI-EXPANDED, SSCI; Timespan=Last 4 weeks

Scopus via Elsevier

TITLE("meta-analysis" OR "systematic review") AND ORIG-LOAD-DATE > 1604275200 AND ORIGLOAD-DATE < 1606867200 AND (LIMIT-TO(DOCTYPE, "ar") OR LIMIT-TO(DOCTYPE, "re"))

Education Collection via ProQuest (added in 30 last days)

MAINSUBJECT.EXACT.EXPLODE("Meta Analysis") OR ab(meta-analysis OR systematic review) OR ti(meta-analysis OR systematic review)

Appendix S4. Data extraction form

Section 1. General characteristics

**Item**	**Response option**
Record ID:	Free text
Systematic review ID (surname year):	Free text
Enter initials of data collector:	Free text
Specify the title of the systematic review:	Free text
Specify the journal:	Free text
Specify the full name of the corresponding author of the systematic review:	Free text
Specify the email address of the corresponding author of the systematic review:	Free text
Specify the country of the corresponding author of the systematic review:	Free text
What was the source of funding for the systematic review?	O Non-profit (e.g. government, university/hospital/research institute, charitable foundation)<br>O For-profit (e.g. pharmaceutical company)<br>O Both non-profit and for-profit<br>O Unclear if the funder is for-profit or non-profit (please specify funder): ___________________<br>O Authors specified there was no funding for the systematic review<br>O Not reported
Did any of the systematic reviewers disclose financial conflicts of interest?	O Conflict of interest present (i.e. at least one systematic reviewer reported a financial conflict of interest of any type)<br>O No conflict of interest (i.e. all systematic reviewers stated they had no financial conflicts of interest)<br>O Missing (i.e. no disclosure statement)
Specify the total number of studies included in the systematic review:	Numeric
Specify the type of participant(s) indicated in the review objective(s) or question(s) (e.g. adults with lung cancer, children with asthma, university students)	Free text
What is the broad ICD-11 category investigated in this systematic review?	O Not applicable<br>O 01 Certain infectious or parasitic diseases<br>O 02 Neoplasms<br>O 03 Diseases of the blood or blood-forming organs

	O 04 Diseases of the immune system<br>O 05 Endocrine, nutritional or metabolic diseases<br>O 06 Mental, behavioural or neurodevelopmental disorders<br>O 07 Sleep-wake disorders<br>O 08 Diseases of the nervous system<br>O 09 Diseases of the visual system<br>O 10 Diseases of the ear or mastoid process<br>O 11 Diseases of the circulatory system<br>O 12 Diseases of the respiratory system<br>O 13 Diseases of the digestive system<br>O 14 Diseases of the skin<br>O 15 Diseases of the musculoskeletal system or connective tissue<br>O 16 Diseases of the genitourinary system<br>O 17 Conditions related to sexual health<br>O 18 Pregnancy, childbirth or the puerperium<br>O 19 Certain conditions originating in the perinatal period<br>O 20 Developmental anomalies<br>O 21 Symptoms, signs or clinical findings, not elsewhere classified<br>O 22 Injury, poisoning or certain other consequences of external causes<br>O 23 External causes of morbidity or mortality<br>O 24 Factors influencing health status or contact with health services
Specify the type of intervention(s) indicated in the review objective(s) or question(s) (e.g. inhaled corticosteroids, provision of charity or welfare, use of bystander programs, reduction in class size) [Select all that apply]:	☐ Health (i.e. any intervention designed to improve “health” defined as a state of complete physical, mental and social well-being and not merely the absence of disease or infirmity)<br>☐ Behavioural (i.e. any intervention designed to increase useful behaviours and reduce or eliminate harmful behaviours, such as modelling, prompting, reinforcement, or shaping behaviours)<br>☐ Educational (i.e. any intervention designed to provide students with the support needed to acquire the skills being taught by an educational system)<br>☐ Social (i.e. any intervention of a government or an organization in social affairs, such as provision of charity or social welfare as a means to alleviate social and economic problems of people facing financial difficulties; provision of safety regulations for employment and products; delivery of food aid, food bank or recovery missions to regions or countries negatively affected by an event; securing workers’ rights through gradualism in institutions, private education and unions)
Is/are the intervention intervention(s) indicated in the review objective(s) or question(s) pharmacological or non-pharmacological?	O Pharmacological

	O Non-pharmacological (i.e. any intervention that does not involve drugs, e.g. device, vitamin, diet, psychotherapy, physical therapy, welfare, organization change, education)<br>O Both pharmacological and non-pharmacological
Notes	Free text

Section 2. Transparency characteristics

Item	Response option
Did the authors state that the systematic review was reported in accordance with a reporting guideline (e.g. PRISMA, MOOSE, MECIR, MARS, ROSES)?	O Yes<br>O No
Is a protocol or registration record for the systematic review cited within the review?	O Both a protocol and registration record are cited<br>O Only a protocol is cited<br>O Only a registration record is cited<br>O Neither are cited
Did the authors specify any eligibility criteria for the following components of the review question [Select all that apply]?	☐ Participants<br>☐ Interventions/exposures<br>☐ Comparators<br>☐ Outcomes
Did the authors make a statement regarding eligibility of studies based on study design?	O Yes<br>O No
[If “Yes” to the previous question] Specify the type of study design(s) eligible for inclusion in the systematic review:	O Randomised trial<br>O Non-randomised study (e.g. cohort study, case-control study)<br>O Both randomised trials and non-randomised studies
How many bibliographic databases were searched? [note: trials registers (e.g. WHO ICTRP, ClinicalTrials.gov) are not applicable to this question]	Numeric
Copy and paste verbatim the list of databases searched:	Free text
Was the interface or platform through which the bibliographic database was searched reported for any database (e.g. did the authors specify that MEDLINE was searched via Ovid, or that CINAHL was searched via EBSCOhost)?	O Fully – interface or platform reported for all databases<br>O Partially – interface or platform reported for only some databases<br>O No – not reported for any database

Were the years of coverage reported for any bibliographic database searched?	O Fully – both start and end exact dates were reported [Select this option if both dates are reported, or if reviewers placed no limit/restriction on the start date and reported the date the search was conducted]<br>O Partially – both start and end dates were reported, but not as exact dates (e.g. MM/YY or WW/MM)<br>O Partially – only the start date or only the end date (e.g. "we searched all databases on 23 May 2019" or "we searched all databases from inception to current")<br>O Not reported
Did the authors specify the exact date when each bibliographic database was last searched?	O Yes<br>O No
Was the full search strategy or search terms used in the bibliographic databases reported?	O Yes – Full Boolean search logic was reported for all databases<br>O Yes – Full Boolean search logic was reported for only some databases<br>O Yes – Only main index terms (e.g. MeSH terms) used in the search strategy were reported<br>O Yes – Only free text terms used in the search strategy were reported<br>O No – Authors did not report any search strategy or search terms
Did the authors report searching a trials register (e.g. ClinicalTrials.gov, WHO International Clinical Trials Registry Platform)?	O Yes<br>O No
Did the authors report searching any other electronic source (e.g. Google Scholar, organisation websites, journal websites)?	O Yes<br>O No
If yes to the previous question, was the search logic that was used to search each of these sources reported?	O Fully – Search logic reported for all these sources<br>O Partially – Search logic reported for only some of these sources<br>O No – Search logic not reported for any of these sources
What method of study selection did the authors report using?	O All identified studies screened by at least two authors<br>O All identified studies screened by one author, with a sample screened by another<br>O At least two authors were involved in either titles/abstracts or full-text screening, but authors did not specify for the remaining step

	O All identified studies screened by only one author<br>O Different methods were applied for titles/abstracts and full-text screening<br>O Not reported/unclear
What method of data extraction did the authors report using?	O All data extracted by at least two authors<br>O All data extracted by one author, with verification by another<br>O All data extracted by only one author<br>O Other (please specify): ____________________<br>O Not reported/Unclear
What method of risk of bias (or quality) assessment did the authors report using?	O All included studies assessed by at least two authors<br>O All included studies assessed by one author, with verification by another<br>O All included studies assessed by only one author<br>O Other (please specify): ____________________<br>O Not reported/Unclear
Did the authors present data on risk of bias (or quality) for each item on a per-study basis (e.g. presented a table or figure displaying how each study was rated on each of the risk of bias (or quality) items)?	O Yes<br>O No
Which statistical software was used to perform meta-analyses [Select all that apply]?	☐ R<br>☐ Stata<br>☐ RevMan<br>☐ Comprehensive Meta-Analysis (CMA)<br>☐ SAS<br>☐ SPSS<br>☐ Other (please specify)<br>☐ Not reported
Did the authors report which statistical package(s) were used (e.g. metan in Stata, metafor in R) and the version number of the package(s)?	O Both the statistical package(s) and software version number(s) were reported<br>O Only the statistical package(s) were reported

	O Only the software version number(s) were reported<br>O Neither were reported
Did the authors report the total number of records identified through all searches?	O Yes<br>O No
Did the authors report the number of records identified through each database/source (e.g. indicated that the search of MEDLINE identified 1000 records, Embase identified 1300 records, CENTRAL identified 750 records, etc.)?	O Yes<br>O No
Did the authors cite any full text articles that were screened and excluded from the review?	O Yes<br>O No
**Answer the following questions for only one meta-analysis per review (the index meta-analysis)**<br>**Select first reported meta-analysis. Note that the first meta-analysis may be identified from the Abstract or Results section of the review, depending on where it is first reported in the publication.**	
What is the outcome domain (e.g. all-cause mortality, anxiety)?	Text
How many studies were included in the meta-analysis?	Numeric
What was the effect measure for the meta-analysis?	O Risk ratio<br>O Odds ratio<br>O Hazard ratio<br>O Risk difference<br>O Mean difference<br>O Standardized mean difference<br>O Other (please specify): ____________________<br>O Not reported

Did the authors describe any methods required to prepare the data for the meta-analysis, such as handling of missing summary statistics, or data conversions?	O Yes<br>O No
Did the authors specify the meta-analysis model used (e.g. fixed-effect, random-effects)? [Note: such information may appear in the Methods section or on the forest plot]	O Yes<br>O No
Did the authors specify the meta-analysis method used (e.g. Mantel-Haenszel, inverse-variance)? [Note: such information may appear in the Methods section or on the forest plot]	O Yes<br>O No
Did the authors specify the between-study (heterogeneity) variance estimator used (e.g. DerSimonian and Laird, restricted maximum likelihood (REML)? [Note: Such information may appear in the Methods section or on the forest plot. Only answer as "Yes" if the estimator was reported explicitly. Answer as "No" if the estimator was not reported explicitly, even if you can guess the estimator from the software used (e.g. because the software implements only one option)]	O Yes<br>O No<br>O Not applicable – not a random-effects meta-analysis
Were summary statistics for each included study reported in a table, figure, or text? By "summary statistics", we mean the mean, standard deviation and sample size for continuous outcomes, or the number of events and sample size for binary outcomes.	O Yes<br>O No
Were effect estimates and measures of precision of each included study reported in a table, figure, or text? [Notes: By "effect estimate", we mean a point estimate of the intervention effect, such as a mean difference for continuous outcomes, or a risk ratio, odds ratio or risk difference for binary outcomes. By "measure of precision", we mean a standard error or confidence interval.]	O Yes<br>O No
Is there a data or code availability statement in the paper?	O Yes<br>O No

If yes to the previous question, copy and paste the data and code statement(s) verbatim:	Free text
Have the authors made any of the following publicly accessible, either as a supplementary file or file uploaded to a general-purpose repository (e.g. Open Science Framework, Zenodo, GitHub) or institutional repository? [Select all that apply. Only select each if you are able to locate the relevant material, after searching all supplementary materials or links to shared materials]	☐ Template data collection form(s)<br>☐ File(s) containing (unprocessed) data extracted from included studies<br>☐ File(s) indicating any necessary data conversions performed<br>☐ File(s) containing data used in all analyses (e.g. Microsoft Excel or CSV spreadsheet, or RevMan file containing all study effect estimates included in meta-analyses)<br>☐ Analytic code used to generate results (i.e. the sequence of commands used within a software package to manage and analyse data)<br>☐ File(s) containing citations of all records that were screened and excluded<br>☐ Metadata which describes the contents of the shared file(s) to aid interpretation and reuse (e.g. a file with complete descriptions of variable names, or README files describing each file shared)
If yes to any of the materials selected, how was the material shared publicly? [Select all that apply]	O Uploaded as a supplementary file on the journal website<br>O Uploaded to a general-purpose open-access repository (e.g. Open Science Framework, Zenodo, GitHub) (please specify)<br>O Uploaded to an institutional repository<br>O Uploaded to my personal website<br>O Other (please specify): ____________________
If yes to any of the materials selected, are any of the data file(s) associated with a persistent identifier (e.g. DOI)?	O Yes<br>O No<br>O Unsure
If yes to any of the materials selected, was a license applied to any of the data file(s) (e.g. CC BY, CC BY-NC)?	O Yes<br>O No<br>O Unsure
Notes	Free text

Section 3. Journal characteristics.

1. Journal name (Abbreviated)	Free text
2. Number of studies published under this journal	Numeric
3. Does this journal only publish evidence syntheses (reviews, meta-analyses and their protocols)?	O Yes<br>O No
**"Instruction for Authors" Section**	
4. Does this journal indicate a data or code sharing policy (or both) under instructions to authors or submission guidelines?	O Yes – The journal encourages or mandates that authors of all research articles (or systematic reviews in particular) share their data or analytic code when they submit an article, as specified in the journal instructions to authors or submission guidelines<br>O No - No such policy can be located in the journal instructions to authors or submission guidelines
5. (If yes to Q4) Copy policy text verbatim	Free text
6. (If yes to Q4) What is the expectation for authors regarding sharing of data and materials (including analytic code)?	O Encouraged: both sharing of data and/or analytic code and the Data Availability Statement are not mandatory for publication by the journal<br>O Expected: sharing data and/or analytic code is not mandatory for publication of systematic reviews, but a Data Availability Statement, which contains links to shared data or reasons for not sharing data, must be provided.<br>O Mandatory: sharing data and/or analytic code is a condition of publication of systematic reviews by the journal;

Appendix S5. List of journals and their policies

Journal	Evidence synthesis journal	Data policy present	Type of data policy	Publisher	Earliest recorded date of journal's data policy *	Earliest recorded date of publisher's data policy **
Acad Emerg Med	No	Yes	Encouraged	Wiley OBO SAEM	14-Sep-21	14-Sep-17
Accid Anal Prev	No	Yes	Encouraged	Elsevier	8-Aug-20	-
Acta Anaesthesiol Scand	No	Yes	Encouraged	Wiley-Blackwell	30-May-21	14-Sep-17
Acta Diabetol	No	Yes	Encouraged	Springer	28-Jul-20	-
Acta Neurol Scand	No	Yes	Expected	Wiley-Blackwell	27-May-19	-
Acta Ophthalmol	No	Yes	Encouraged	Wiley-Blackwell	29-Nov-19	-
Acta Psychiatr Scand	No	Yes	Expected	Wiley-Blackwell	5-May-19	-
Addiction	No	Yes	Encouraged	Wiley-Blackwell	4-May-19	-
Adv Ther	No	No	-	Springer	-	-
Adv Nutr (Bethesda, Md)	No	No	-	ASN	-	-
Aids	No	No	-	Lippincott W&W	-	-
Aliment Pharmacol Ther	No	Yes	Expected	Wiley-Blackwell	5-May-19	-
Am Heart J	No	Yes	Encouraged	Elsevier	30-Jan-19	-
Am J Cardiol	No	Yes	Encouraged	Elsevier	26-Jan-20	-
Am J Mens Health	No	No	-	SAGE	-	-
Am J Sports Med	No	No	-	SAGE	-	-
Ann Palliat Med	No	No	-	Mary Ann Liebert	-	-
Ann Transl Med	No	No	-	AME Publishing	-	-
Ann Surg	No	No	-	Lippincott W&W	-	-
Ann Roy Coll Surg	No	No	-	Royal College of Surgeons of England	-	-
Antioxidants (Basel)	No	Yes	Mandatory	MDPI	12-May-21	10-Oct-20
Arch Public Health	No	Yes	Expected	BMC	n/a	12-Jun-16
Arthritis Rheumatol	No	Yes	Mandatory	Wiley & Sons	21-Oct-20	-
Arthrosc Sports Med Rehabil	No	Yes	Encouraged	Elsevier	1-Aug-20	-
Attach Hum Dev	No	Yes	Expected	Taylor & Francis	n/a	13-Feb-18
Aust Dent J	No	Yes	Encouraged	Wiley-Blackwell	8-Mar-22	14-Sep-17
Aust J Prim Health	No	Yes	Expected	CSIRO	4-Jul-22	4-Sep-17
Autoimmun Rev	Yes	No	-	Elsevier	-	-
Biomed Res Int	No	No	-	Hindawi	-	-
Bioscience Rep	No	Yes	Expected	Portland Press	28-Sep-20	-
Birth Defects Res	No	Yes	Expected	Wiley & Sons	17-Jul-19	-
BJOG	No	Yes	Expected	Wiley-Blackwell	19-Jun-20	-
BJS Open	No	Yes	Encouraged	Oxford Uni	5-Apr-22	Early 2020
Blood Purif	No	Yes	Encouraged	Karger AG	14-Sep-20	-
BMC Musculoskelet Disord	No	Yes	Expected	BMC	n/a	12-Jun-16
BMC Psychiatry	No	Yes	Expected	BMC	n/a	12-Jun-16
BMC Urol	No	Yes	Expected	BMC	n/a	12-Jun-16
BMJ	No	Yes	Expected	BMJ	11-Jan-18	-
BMJ Open	No	Yes	Expected	BMJ	30-Jan-19	-
BMJ Open Sport Exerc Med	No	Yes	Expected	BMJ	15-Dec-19	-
BMJ Support Palliat Care	No	Yes	Expected	BMJ	7-Jan-19	-

Journal	Evidence synthesis journal	Data policy present	Type of data policy	Publisher	Earliest recorded date of journal's data policy *	Earliest recorded date of publisher's data policy **
Bone Rep	No	Yes	Encouraged	Elsevier	24-Dec-19	-
Br J Anaesth	No	Yes	Encouraged	Elsevier	24-Dec-19	-
Br J Sports Med	No	Yes	Expected	BMJ	11-Jan-18	-
Braz Oral Res	No	No	-	Sao Paolo Uni	-	-
Can J Cardiol	No	Yes	Encouraged	Elsevier	25-Sep-20	-
Can Respir J	No	No	-	Hindawi	-	-
Cancer	No	Yes	Encouraged	Wiley & Sons	22-Sep-20	-
Cancers	No	Yes	Mandatory	MDPI	25-Nov-20	10-Oct-20
Cardiol J	No	Yes	Encouraged	Via Medica	16-Jul-20	-
Cardiovasc Drugs Ther	No	Yes	Encouraged	Springer	30-Jul-21	5-Jul-16
Cardiovasc Interv Ther	No	No	-	Springer	-	-
Cardiovasc Revasc Med	No	Yes	Encouraged	Elsevier	10-Jun-21	4-Sep-17
Catheter Cardiovasc Interv	No	No	-	Wiley-Liss	-	-
Clin Cardiol	No	No	-	Wiley-Blackwell	-	-
Clin Infect Dis	No	Yes	Encouraged	Oxford Uni	22-May-22	Early 2020
Clin Microbiol Infect	No	No	-	Elsevier	-	-
Clin Nutr	No	Yes	Encouraged	Elsevier	7-Sep-20	-
Clin Nutr ESPEN	No	Yes	Encouraged	Elsevier	20-Sep-20	-
Clin Oral Investig	No	Yes	Encouraged	Springer	24-Sep-20	-
Clin Rehabil	No	No	-	SAGE	-	-
Clin Transl Oncol	No	Yes	Encouraged	Springer	28-Nov-21	5-Jul-16
Clin Neuropharmacol	No	No	-	Lippincott W&W	-	-
Cochrane Database Syst Rev	Yes	Yes	Mandatory	Wiley & Sons	1-Apr-14	-
Complement Ther Clin Pract	No	Yes	Encouraged	Elsevier	22-Oct-20	-
Complement Ther Med	No	Yes	Encouraged	Elsevier	7-Sep-20	-
Crit Rev Oncol Hematol	Yes	Yes	Encouraged	Elsevier	4-Apr-20	-
Dan Med J	No	No	-	Almindelige Danske Laegeforening	-	-
Dermatol Ther	No	Yes	Expected	Wiley-Blackwell	18-Oct-20	-
Diabetes Metab Syndr: Clin Res Rev	No	Yes	Encouraged	Elsevier	n/a	4-Sep-17
Diabetes Ther	No	No	-	Springer	-	-
Diagn Interv Radiol	No	No	-	Turkish Society of Radiology	-	-
Disabil Rehabil	No	Yes	Encouraged	Taylor & Francis	n/a	13-Feb-18
Drug Saf	No	Yes	Encouraged	Springer	9-Aug-20	-
EClinicalMedicine	No	Yes	Expected	Lancet	21-Sep-20	-
Eur Heart J	No	Yes	Expected	Oxford Uni	29-Mar-20	-
Eur Heart J Cardiovasc Pharmacother	No	Yes	Expected	Oxford Uni	15-May-20	-
Eur J Anaesth	No	No	-	Lippincott W&W	-	-
Eur J Cancer	No	Yes	Encouraged	Elsevier	14-Aug-20	-
Eur J Clin Microbiol Infect Dis	No	No	-	Springer	-	-
Eur J Hosp Pharm	No	Yes	Expected	European Association of Hospital Pharmacists	4-Jan-19	-
Eur J Nutr	No	Yes	Encouraged	Springer	19-May-20	-
Eur J Ophthalmol	No	No	-	Wichtig	-	-

Journal	Evidence synthesis journal	Data policy present	Type of data policy	Publisher	Earliest recorded date of journal's data policy *	Earliest recorded date of publisher's data policy **
Eur J Pediatr	No	Yes	Encouraged	Springer	10-Aug-20	-
Eur J Pediatr Surg	No	No	-	Thieme	-	-
Eur Radiol	No	No	-	Springer	-	-
Eur Rev Med Pharmacol Sci	Yes	No	-	Verduci Editore	-	-
Eur Spine J	No	Yes	Encouraged	Springer	30-Aug-20	-
Eur J Integr Med	No	Yes	Encouraged	Elsevier	7-Sep-20	-
Evid Based Complement Alternat Med	No	No	-	Hindawi	-	-
Exp Gerontol	No	Yes	Encouraged	Elsevier	7-Sep-20	-
Food Funct	No	No	-	Royal Society of Chemistry	-	-
Foot Ankle Surg	No	Yes	Encouraged	Elsevier	5-Aug-20	-
Front Oncol	No	No	-	Frontiers Media	-	-
Front Pharmacol	No	No	-	Frontiers Media	-	-
Front Psychiatry	No	No	-	Frontiers Media	-	-
Front Surg	No	No	-	Frontiers Media	-	-
Gastroenterol Hepatol	No	Yes	Encouraged	Elsevier	26-Aug-16	-
Gen Thorac Cardiovasc Surg	No	No	-	Springer	-	-
Gerodontology	No	Yes	Encouraged	Wiley-Blackwell	8-Mar-22	14-Sep-17
Hepatology	No	Yes	Mandatory	Wiley & Sons	n/a	14-Sep-17
Horm Mol Biol Clin Investig	No	No	-	de Gruyter	-	-
Hum Reprod Update	No	Yes	Expected	Oxford Uni	17-May-20	-
Indian J Med Microbiol	No	Yes	Encouraged	Elsevier	29-Nov-21	4-Sep-17
Indian J Ophthalmol	No	No	-	Wolters Kluwer	-	-
Indian J Surg	No	Yes	Encouraged	Springer	7-Aug-20	-
Injury	No	Yes	Encouraged	Elsevier	8-Aug-20	-
Int Braz J Urol	No	No	-	Brazilian Society of Urology	-	-
Int Breastfeed J	No	Yes	Expected	BMC	n/a	12-Jun-16
Int J Clin Pract	No	Yes	Encouraged	Wiley-Blackwell / Hindawi (22 Sep 2021 onwards)	30-Mar-22	14-Sep-17
Int J Endocrinol	No	No	-	Hindawi	-	-
Int J Environ Res Public Health	No	Yes	Mandatory	MDPI	29-Oct-20	10-Oct-20
Int J Paediatr Dent	No	Yes	Encouraged	Wiley-Blackwell	8-Mar-22	14-Sep-17
Int J Rehabil Res	No	No	-	Lippincott W&W	-	-
Int J Vitam Nutr Res	No	No	-	Hogrefe	-	-
Int Wound J	No	Yes	Encouraged	Wiley-Blackwell	18-Aug-21	14-Sep-17
Integr Cancer Ther	No	Yes	Mandatory	SAGE	24-Apr-20	-
Integr Med Res	No	Yes	Encouraged	Elsevier	15-Jan-19	-
Iran J Microbiol	No	No	-	Teheran University of Medical Sciences	-	-
J Adolesc	No	Yes	Encouraged	Elsevier / Wiley (as of 1 Jan 2022)	15-Oct-18	-
J Affect Disord	No	Yes	Encouraged	Elsevier	22-Oct-20	-
J Altern Complement Med	No	Yes	Encouraged	Mary Ann Liebert	21-Sep-20	-
J Am Med Dir Assoc	No	Yes	Encouraged	Elsevier	24-Oct-19	-
J Arthroplasty	No	Yes	Encouraged	Elsevier	3-Feb-21	4-Sep-17
J Bodyw Mov Ther	No	No	-	Churchill Livingstone	-	-

Journal	Evidence synthesis journal	Data policy present	Type of data policy	Publisher	Earliest recorded date of journal's data policy *	Earliest recorded date of publisher's data policy **
J Cardiovasc Pharmacol	No	No	-	Lippincott W&W	-	-
J Clin Med	No	Yes	Mandatory	MDPI	11-Nov-20	10-Oct-20
J Clin Neurosci	No	Yes	Encouraged	Elsevier	29-Aug-17	-
J Clin Pharm Ther	No	No	-	Wiley-Blackwell	-	-
J Coll Physicians Surg Pak	No	No	-	College of Physicians and Surgeons Pakistan	-	-
J Consult Clin Psychol	No	Yes	Expected	APA	8-Aug-17	-
J Craniofac Surg	No	No	-	Lippincott W&W	-	-
J Crit Care	No	Yes	Encouraged	Elsevier	7-Sep-20	-
J Dent Anesth Pain Med	No	No	-	Korean Dental Society of Anesthesiology	-	-
J Diabetes Investig	No	No	-	Blackwell Asia	-	-
J Emerg Med	No	Yes	Encouraged	Elsevier	4-Sep-18	-
J Food Biochem	No	Yes	Expected	Wiley-Blackwell	n/a	14-Sep-17
J Formos Med Assoc	No	Yes	Encouraged	Excerpta Medica	11-Nov-19	-
J Gastroenterol Hepatol	No	Yes	Encouraged	Wiley-Blackwell	4-May-19	-
J Gynecol Oncol	No	No	-	Korean Society of Gynecologic Oncology and Colposcopy	-	-
J Hepato-Biliary-Pancreat Sci	No	Yes	Encouraged	Wiley-Blackwell	n/a	14-Sep-17
J Herb Med	No	Yes	Encouraged	Elsevier	11-Aug-20	-
J Int AIDS Soc	No	Yes	Expected	Wiley & Sons	25-Jul-20	-
J Int Med Res	No	Yes	Mandatory	SAGE	26-Jun-20	-
J Intensive Care	No	Yes	Expected	BMC	n/a	12-Jun-16
J Interv Cardiol	No	No	-	Wiley-Blackwell	-	-
J Invest Surg	No	Yes	Encouraged	Taylor & Francis	15-Jun-22	13-Feb-18
J Matern Fetal Neonatal Med	No	Yes	Encouraged	Taylor & Francis	n/a	13-Feb-18
J Med Internet Res	No	Yes	Encouraged	JMIR	5-Aug-20	-
J Minim Invasive Gynecol	No	Yes	Encouraged	Elsevier	7-Sep-20	-
J Obstet Gynaecol	No	Yes	Encouraged	Taylor & Francis	n/a	13-Feb-18
J Ophthalmol	No	No	-	Hindawi	-	-
J Oral Biol Craniofac Res	No	Yes	Encouraged	Elsevier	5-Sep-20	-
J Oral Implantol	No	No	-	Allen Press	-	-
J Orthop Surg Res	No	Yes	Expected	BMC	n/a	12-Jun-16
J Pediatr Urol	No	Yes	Encouraged	Elsevier	21-Aug-20	-
J Pharm Sci	No	No	-	Wiley & Sons	-	-
J Prosthet Dent	No	Yes	Encouraged	Elsevier	23-Aug-22	4-Sep-17
J Rehabil Med	No	No	-	Foundation for Rehabilitation Information	-	-
J Shoulder Elbow Surg	No	No	-	Mosby	-	-
J Surg Res	No	Yes	Encouraged	Elsevier	9-Mar-22	4-Sep-17
J Tradit Chin Med	No	No	-	Journal of Traditional Chinese Medicine	-	-
J Vasc Surg	No	No	-	Mosby	-	-
J Wound Care	No	No	-	MA Healthcare	-	-
Jama	No	No	-	AMA	-	-
JAMA Psychiatry	No	No	-	AMA	-	-

Journal	Evidence synthesis journal	Data policy present	Type of data policy	Publisher	Earliest recorded date of journal's data policy *	Earliest recorded date of publisher's data policy **
JMIR Serious Games	No	No	-	JMIR	-	-
J Clin Anesth	No	Yes	Encouraged	Elsevier	27-Jul-20	-
J Endourol	No	No	-	Mary Ann Liebert	-	-
J Hosp Infect	No	Yes	Encouraged	Elsevier	8-Mar-21	4-Sep-17
Knee Surg Sports Traumatol Arthrosc	No	Yes	Encouraged	Springer	n/a	5-Jul-16
Korean J Intern Med	No	No	-	Korean Association of Internal Medicine	-	-
Life (Basel)	No	Yes	Mandatory	MDPI	21-May-21	10-Oct-20
Medicine (Baltimore)	No	Yes	Expected	Lippincott W&W	8-Mar-20	-
Minerva Anestesiol	No	No	-	Minerva Medica	-	-
Neurochirurgie	No	Yes	Encouraged	Elsevier	9-Sep-20	-
Neurology	No	Yes	Mandatory	Lippincott W&W	26-Jul-20	-
Nurs Crit Care	No	Yes	Encouraged	Wiley-Blackwell	9-Jun-21	14-Sep-17
Nutrients	No	Yes	Mandatory	MDPI	25-Oct-20	10-Oct-20
Obes Rev	Yes	Yes	Encouraged	Wiley-Blackwell	6-May-19	-
Obesity (Silver Spring)	No	No	-	Wiley-Blackwell	-	-
Oper Dent	No	No	-	Indiana University School of Dentistry	-	-
Oral Health Prev Dent	No	No	-	Quintessence Publishing	-	-
Orthop Traumatol Surg Res	No	Yes	Encouraged	Elsevier	7-Aug-20	-
PACE-Pacing Clin Electrophysiol	No	No	-	Wiley-Blackwell	-	-
Pediatr Surg Int	No	Yes	Encouraged	Springer	26-Nov-20	5-Jul-16
Pediatrics	No	No	-	American Academy of Pediatrics	-	-
Pharmacol Res	No	Yes	Encouraged	Elsevier	11-Aug-20	-
Phys Ther	No	Yes	Encouraged	Oxford Uni	29-Mar-20	-
Physiother Theory Pract	No	Yes	Encouraged	Taylor & Francis	23-Feb-22	13-Feb-18
Phytother Res	No	Yes	Expected	Wiley & Sons	17-Aug-21	14-Sep-17
PLoS One	No	Yes	Mandatory	PLOS	8-May-15	-
Pol Intern Med	No	No	-	Medycyna Praktyczna	-	-
Porto Biomed J	No	No	-	Elsevier	-	-
Postgrad Med J	No	Yes	Expected	BMJ	11-Jan-18	-
Prog Neuropsychopharmacol Biol Psychiatry	No	Yes	Encouraged	Elsevier	22-Oct-20	-
Psychiatry Res	No	Yes	Encouraged	Elsevier	21-Nov-18	-
Psychol Med	No	No	-	Cambridge Uni	-	-
Psychol Sport Exerc	No	No	-	Human Kinetics	-	-
Reg Anesth Pain Med	No	Yes	Expected	BMJ	11-Jan-18	-
Reprod Biomed Online	No	Yes	Encouraged	Elsevier	30-Oct-20	-
Scand J Gastroenterol	No	Yes	Encouraged	Taylor & Francis	n/a	13-Feb-18
Schizophr Res	No	No	-	Elsevier	-	-
Sci Rep	No	No	-	Nature	-	-
Signa Vitae	No	No	-	Pharmamed Mado	-	-
Sleep Breath	No	Yes	Expected	Springer	11-Jul-22	5-Jul-16
Spine	No	No	-	Lippincott W&W	-	-
Sports Med	No	Yes	Encouraged	Springer	10-Aug-20	-

Journal	Evidence synthesis journal	Data policy present	Type of data policy	Publisher	Earliest recorded date of journal's data policy *	Earliest recorded date of publisher's data policy **
Steroids	No	Yes	Encouraged	Elsevier	24-Oct-20	-
Surg Obes Relat Dis	No	Yes	Encouraged	Elsevier	19-Mar-19	-
Surgery	No	Yes	Encouraged	Elsevier	24-Aug-20	-
Tech Coloproctol	No	No	-	Springer	-	-
Thromb Res	No	Yes	Encouraged	Elsevier	20-May-20	-
Transl Androl Urol	No	No	-	AME Publishing	-	-
Updates Surg	No	Yes	Encouraged	Springer	n/a	5-Jul-16
Urol J	No	Yes	Encouraged	Elsevier OBO Urology and Nephrology RC	14-Jan-21	4-Sep-17
Vaccine	No	Yes	Encouraged	Elsevier	19-Apr-20	-
Work	No	No	-	IOS Press	-	-
World J Surg	No	Yes	Encouraged	Springer	22-Jun-20	-
World Neurosurg	No	Yes	Encouraged	Elsevier	18-Apr-21	4-Sep-17
Wound Manag Prev	No	No	-	HMP Communications	-	-
Wound Repair Regen	No	Yes	Mandatory	Wiley-Blackwell	n/a	14-Sep-17
Z Rheumatol	No	No	-	Wiley-Blackwell	-	-

Note:

** For journals with a data policy, we used the Wayback Machine tool (https://web.archive.org/) to search for web archives of the Author Instructions page on each journal's website, and recorded the type of data policy based on the version published prior to 1 November 2020.*

***If no archived version of the Author Instructions page prior to 1 November 2020 was found, we recorded the type of data policy based on the earliest version available and verified that the publisher announced the rollout of a universal data policy prior to 1 November 2020 (which supports our assumption that the data policy of the journal had already been in place before 1 November 2020).*

Appendix S6: Comparison between reviews of health interventions in 2014 vs 2020

Item	Median (IQR) or Frequency (%)	
	**2020*** **(N=294)**	**2014** **(N=110)**
Number of databases searched	4 (3-5)	4 (3-5)
Number of studies included in review	13 (8-21)	13 (7-23)
Number of studies in index meta-analysis	6 (4-10)	6 (3-11)
Country of corresponding author		
China (2014) | China (2020)	95/294 (32)	23/110 (21)
UK (2014) | USA (2020)	29/294 (10)	17/110 (15)
Canada (2014) | UK (2020)	24/294 (8)	15/110 (14)
Other	146/294 (50)	55/110 (50)
Cochrane reviews	8/294 (3)	32/110 (29)
Source of funding		
Author reported funding	115/294 (39)	60/110 (55)
Author reported no funding	95/294 (32)	12/110 (11)
Not reported	84/294 (29)	38/110 (35)
Conflict of interest declared	276/294 (94)	103/110 (94)
Area(s) of intervention		
Pharmacological	102/294 (35)	55/110 (50)
Non-pharmacological	183/294 (62)	43/110 (39)
Both	9/294 (3)	12/110 (11)
ICD-11 category investigated		
Endocrine, nutritional or metabolic diseases (2020)<br>Diseases of the digestive system (2014)	36/294 (12)	14/110 (13)
Diseases of the digestive system (2020)<br>Infectious or parasitic diseases (2014)	36/294 (12)	13/110 (12)
Diseases of the musculoskeletal system (2020)<br>Diseases of the circulatory system (2014)	35/294 (12)	13/110 (12)
Diseases of the circulatory system (2020)<br>Neoplasms (2014)	30/294 (10)	11/110 (10)
Other	157/294 (53)	59/110 (54)
Citing a reporting guideline	242/294 (82)	32/110 (29)
Protocol/registration record cited		
Both protocol and registration record cited	1/294 (<0.5)	4/110 (4)
Only a protocol cited	12/294 (4)	32/110 (29)
Only a registration record cited	110/294 (37)	2/110 (2)
Neither	171/294 (58)	72/110 (65)
Type(s) of eligible study design stated	272/294 (93)	104/110 (95)
Randomised studies	164/272 (60)	71/110 (65)
Non-randomised studies	20/272 (7)	7/110 (6)
Both	88/272 (32)	32/110 (29)
Dates of coverage of databases reported		
Both start and end dates	134/294 (46)	77/110 (70)
Either start or end date only	152/294 (52)	29/110 (26)
Not reported	8/294 (3)	4/110 (4)
Boolean search strategy reported for at least 1 database	211/294 (72)	60/110 (55)
Trials register searched	63/294 (21)	38/110 (35)

Item	Median (IQR) or Frequency (%)	
	2020* (N=294)	2014 (N=110)
Other electronic sources searched	97/294 (33)	101/110 (92)
Method of study screening reported	228/294 (78)	85/110 (77)
Method of data collection reported	227/294 (77)	84/110 (76)
Method of ROB assessment reported	181/294 (62)	63/95 (66)
Total records retrieved reported	294/294 (100)	91/110 (83)
Software(s) used for meta-analysis		
R	33/294 (11)	7/110 (6)
Stata	73/294 (25)	16/110 (15)
Review Manager	185/294 (63)	80/110 (73)
Comprehensive Meta-Analysis	25/294 (9)	6/110 (5)
SAS	1/294 (<0.5)	2/110 (2)
SPSS	3/294 (1)	4/110 (4)
Other	13/294 (4)	12/110 (11)
Not reported	3/294 (1)	1/110 (1)
Sharing of data or materials used in analyses	19/294 (6)	33/110 (30)
**Index meta-analysis**		
Measure of effect used		
Risk ratio	70/294 (24)	36/110 (33)
Odds ratio	70/294 (24)	20/110 (18)
Hazard ratio	13/294 (4)	6/110 (5)
Risk difference	3/294 (1)	2/110 (2)
Mean difference	76/294 (26)	25/110 (23)
Standardised mean difference	60/294 (20)	10/110 (9)
Other	2/294 (1)	11/110 (10)
Method of data preparation reported	100/294 (34)	17/110 (15)
Meta-analysis model reported (e.g. fixed-effects, random-effects)	289/294 (98)	106/110 (96)
Summary statistics reported for each study	211/294 (72)	79/110 (72)
Effect estimate and measure of precision reported for each study	282/294 (96)	101/110 (92)

**The 2020 sample only includes reviews of health interventions.*

Figure S1: Sensitivity analysis – Frequency of reporting items between systematic reviews published in 2014 and 2020

Reported item		2020	2014	Risk ratio (95% CI)
Citation of a reporting guideline	OA	242/294 (82)	32/110 (29)	2.8 (2.1 to 3.8)
	SA	234/286 (82)	32/78 (41)	2.0 (1.5 to 2.6)
Funding source	OA	210/294 (71)	72/110 (65)	1.1 (0.9 to 1.3)
	SA	204/286 (71)	42/78 (54)	1.3 (1.1 to 1.6)
Conflict of interest	OA	276/294 (94)	103/110 (94)	1.0 (0.9 to 1.1)
	SA	268/286 (94)	71/78 (91)	1.0 (1.0 to 1.1)
Protocol / registration record	OA	123/294 (42)	38/110 (35)	1.2 (0.9 to 1.6)
	SA	115/286 (40)	7/78 (9)	4.5 (2.2 to 9.2)
Eligiblity criteria - Outcomes	OA	292/294 (99)	105/110 (95)	1.0 (1.0 to 1.1)
	SA	284/286 (99)	73/78 (94)	1.1 (1.0 to 1.1)
Eligibility criteria - Study design	OA	272/294 (93)	104/110 (95)	1.0 (0.9 to 1.0)
	SA	264/286 (92)	72/78 (92)	1.0 (0.9 to 1.1)
Dates of coverage of databases	OA	236/294 (80)	77/110 (70)	1.1 (1.0 to 1.3)
	SA	229/286 (80)	48/78 (62)	1.3 (1.1 to 1.6)
Search strategy - Databases	OA	211/294 (72)	60/110 (55)	1.3 (1.1 to 1.6)
	SA	203/286 (71)	28/78 (36)	2.0 (1.5 to 2.7)
Screening method	OA	228/294 (78)	85/110 (77)	1.0 (0.9 to 1.1)
	SA	220/286 (77)	53/78 (68)	1.1 (1.0 to 1.3)
Data collection method	OA	227/294 (77)	84/110 (76)	1.0 (0.9 to 1.1)
	SA	219/286 (77)	53/78 (68)	1.1 (1.0 to 1.3)
ROB assessment method	OA	181/294 (62)	63/95 (66)	0.9 (0.8 to 1.1)
	SA	173/286 (60)	35/63 (56)	1.1 (0.9 to 1.4)
Total records retrieved	OA	294/294 (100)	91/110 (83)	1.2 (1.1 to 1.3)
	SA	286/286 (100)	72/78 (92)	1.1 (1.0 to 1.2)
Meta-analysis model (e.g. fixed-effects, random-effects)	OA	289/294 (98)	106/110 (96)	1.0 (1.0 to 1.1)
	SA	281/286 (98)	74/78 (95)	1.0 (1.0 to 1.1)
Summary statistics per study	OA	211/294 (72)	79/110 (72)	1.0 (0.9 to 1.1)
	SA	203/286 (71)	50/78 (64)	1.1 (0.9 to 1.3)
Effect estimate and measure of precision per study	OA	282/294 (96)	101/110 (92)	1.0 (1.0 to 1.1)
	SA	274/286 (96)	69/78 (88)	1.1 (1.0 to 1.2)
Data preparation method	OA	100/294 (34)	17/110 (15)	2.2 (1.4 to 3.5)
	SA	92/286 (32)	12/78 (15)	2.1 (1.2 to 3.6)
Sharing of data and materials	OA	19/294 (6)	33/110 (30)	0.2 (0.1 to 0.4)
	SA	11/286 (4)	1/78 (1)	2.2 (0.4 to 12.0)

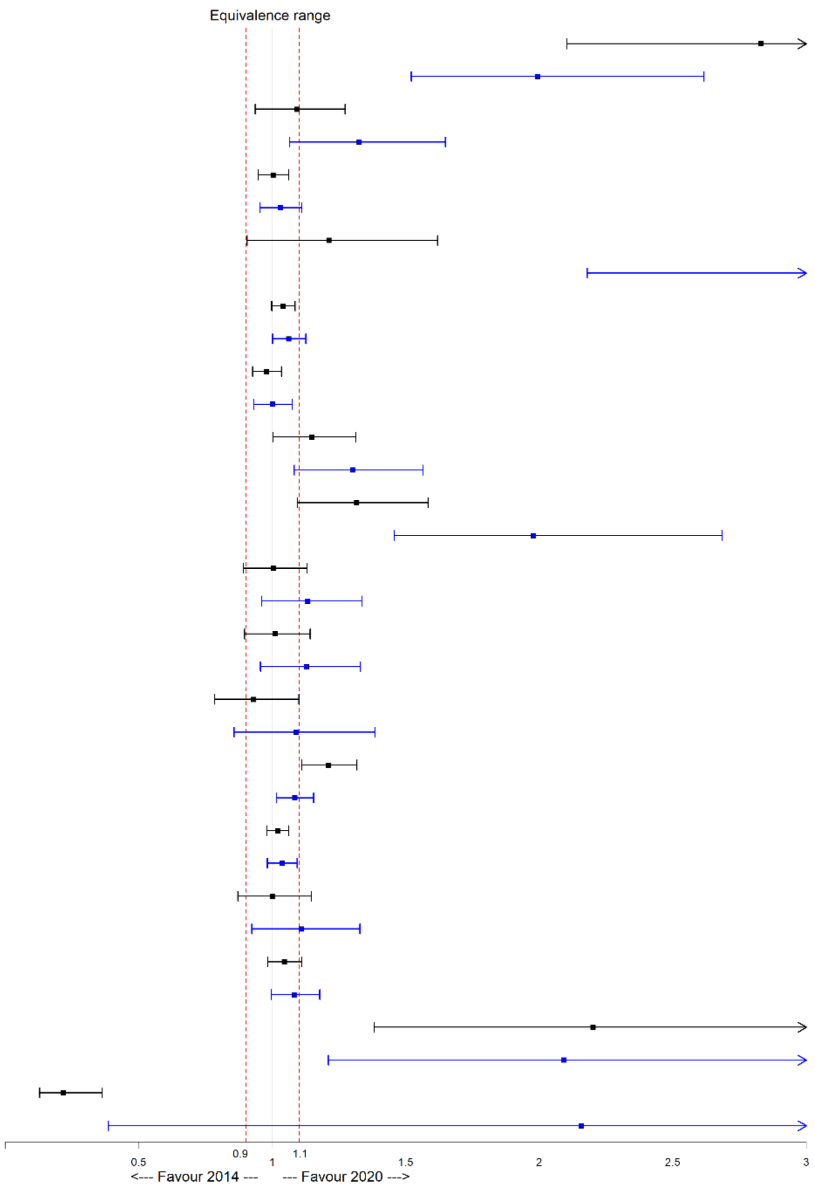

Legend OA Original analysis
SA Sensitivity analysis (excluding Cochrane reviews)

Figure S2A: Sensitivity analysis – Relationship between citation of reporting guidelines and reported items

Reported item		Guideline	No guideline	Risk ratio (95% CI)
Funding source	OA	172/245 (70)	43/55 (78)	0.9 (0.8 to 1.1)
	SA	168/239 (70)	42/53 (79)	0.9 (0.8 to 1.0)
Conflict of interest	OA	226/245 (92)	55/55 (100)	0.9 (0.9 to 1.0)
	SA	221/239 (92)	53/53 (100)	0.9 (0.9 to 1.0)
Protocol / registration record	OA	108/245 (44)	18/55 (33)	1.3 (0.9 to 2.0)
	SA	107/239 (45)	17/53 (32)	1.4 (0.9 to 2.1)
Eligibility criteria - Participants	OA	225/245 (92)	50/55 (91)	1.0 (0.9 to 1.1)
	SA	220/239 (92)	48/53 (91)	1.0 (0.9 to 1.1)
Eligibility criteria - Intervention	OA	241/245 (98)	55/55 (100)	1.0 (1.0 to 1.0)
	SA	236/239 (99)	53/53 (100)	1.0 (1.0 to 1.0)
Eligibility criteria - Comparator	OA	219/245 (89)	48/55 (87)	1.0 (0.9 to 1.1)
	SA	215/239 (90)	46/53 (87)	1.0 (0.9 to 1.2)
Eligiblity criteria - Outcomes	OA	243/245 (99)	55/55 (100)	1.0 (1.0 to 1.0)
	SA	237/239 (99)	53/53 (100)	1.0 (1.0 to 1.0)
Eligibility criteria - Study design	OA	229/245 (93)	49/55 (89)	1.0 (1.0 to 1.2)
	SA	225/239 (94)	47/53 (89)	1.1 (1.0 to 1.2)
Interface used to search databases	OA	94/245 (38)	18/55 (33)	1.2 (0.8 to 1.8)
	SA	91/239 (38)	17/53 (32)	1.2 (0.8 to 1.8)
Dates of coverage of databases	OA	194/245 (79)	47/55 (85)	0.9 (0.8 to 1.1)
	SA	190/239 (79)	46/53 (87)	0.9 (0.8 to 1.0)
Date of last search	OA	62/245 (25)	10/55 (18)	1.4 (0.8 to 2.5)
	SA	57/239 (24)	9/53 (17)	1.4 (0.7 to 2.7)
Search strategy - Databases	OA	178/245 (73)	36/55 (65)	1.1 (0.9 to 1.4)
	SA	172/239 (72)	35/53 (66)	1.1 (0.9 to 1.3)
Search strategy - Other sources	OA	22/115 (19)	2/25 (8)	2.3 (0.6 to 9.0)
	SA	22/110 (20)	2/23 (9)	2.3 (0.6 to 9.0)

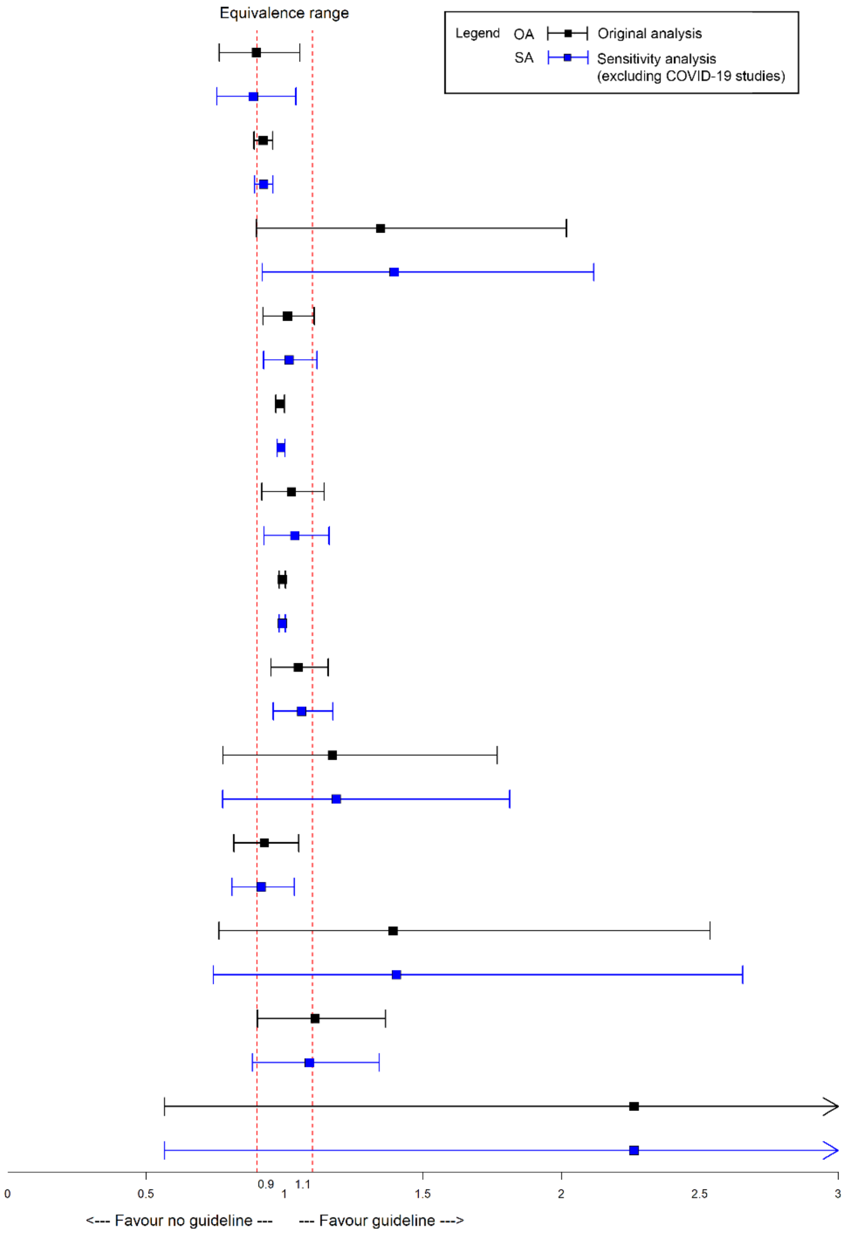

Figure S2B: Sensitivity analysis – Relationship between citation of reporting guidelines and reported items (ctn'd)

Reported item		Guideline	No guideline	Risk ratio (95% CI)
Screening method	OA	195/245 (80)	38/55 (69)	1.2 (1.0 to 1.4)
	SA	191/239 (80)	36/53 (68)	1.2 (1.0 to 1.4)
Data collection method	OA	182/245 (74)	47/55 (85)	0.9 (0.8 to 1.0)
	SA	176/239 (74)	45/53 (85)	0.9 (0.8 to 1.0)
ROB assessment method	OA	154/245 (63)	31/55 (56)	1.1 (0.9 to 1.4)
	SA	148/239 (62)	29/53 (55)	1.1 (0.9 to 1.5)
ROB assessment per study	OA	190/245 (78)	41/55 (75)	1.0 (0.9 to 1.2)
	SA	184/239 (77)	40/53 (75)	1.0 (0.9 to 1.2)
Total records retrieved	OA	245/245 (100)	55/55 (100)	1.0 (1.0 to 1.0)
	SA	239/239 (100)	53/53 (100)	1.0 (1.0 to 1.0)
Records retrieved per database	OA	109/245 (44)	17/55 (31)	1.4 (0.9 to 2.2)
	SA	106/239 (44)	17/53 (32)	1.4 (0.9 to 2.1)
Citing at least 1 excluded articles	OA	56/245 (23)	9/55 (16)	1.4 (0.7 to 2.6)
	SA	54/239 (23)	8/53 (15)	1.5 (0.8 to 3.0)
Data preparation methods	OA	85/245 (35)	16/55 (29)	1.2 (0.8 to 1.9)
	SA	84/239 (35)	16/53 (30)	1.2 (0.7 to 1.8)
Meta-analysis model (e.g. fixed-effects, random-effects)	OA	240/245 (98)	54/55 (98)	1.0 (1.0 to 1.0)
	SA	234/239 (98)	52/53 (98)	1.0 (1.0 to 1.0)
Meta-analysis method (e.g. Mantel-Haenszel, inverse variance)	OA	179/245 (73)	39/55 (71)	1.0 (0.9 to 1.2)
	SA	174/239 (73)	38/53 (72)	1.0 (0.8 to 1.2)
Heterogeneity variance estimator (e.g. DerSimonian Laird)	OA	45/197 (23)	5/38 (13)	1.8 (0.7 to 4.7)
	SA	45/192 (23)	5/36 (14)	1.8 (0.7 to 4.7)
Summary statistics per study	OA	174/245 (71)	41/55 (75)	1.0 (0.8 to 1.1)
	SA	168/239 (70)	39/53 (74)	1.0 (0.8 to 1.1)
Effect estimate and measure of precision per study	OA	234/245 (96)	54/55 (98)	1.0 (0.9 to 1.0)
	SA	228/239 (95)	52/53 (98)	1.0 (0.9 to 1.0)
Sharing of data and materials	OA	18/245 (7)	2/55 (4)	1.7 (0.4 to 6.7)
	SA	18/239 (8)	2/53 (4)	1.7 (0.4 to 6.7)

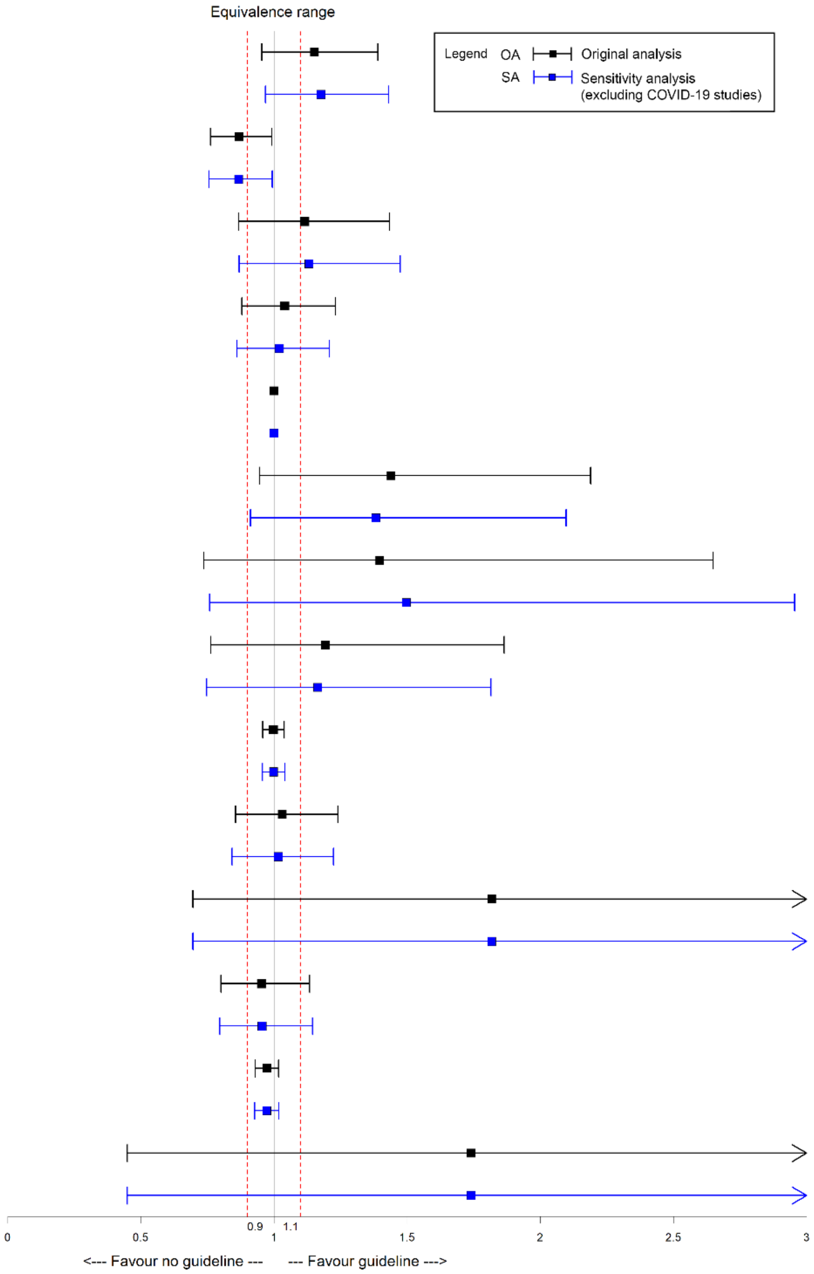

Figure S3: Relationship between journal's presence of a data sharing policy and reported items

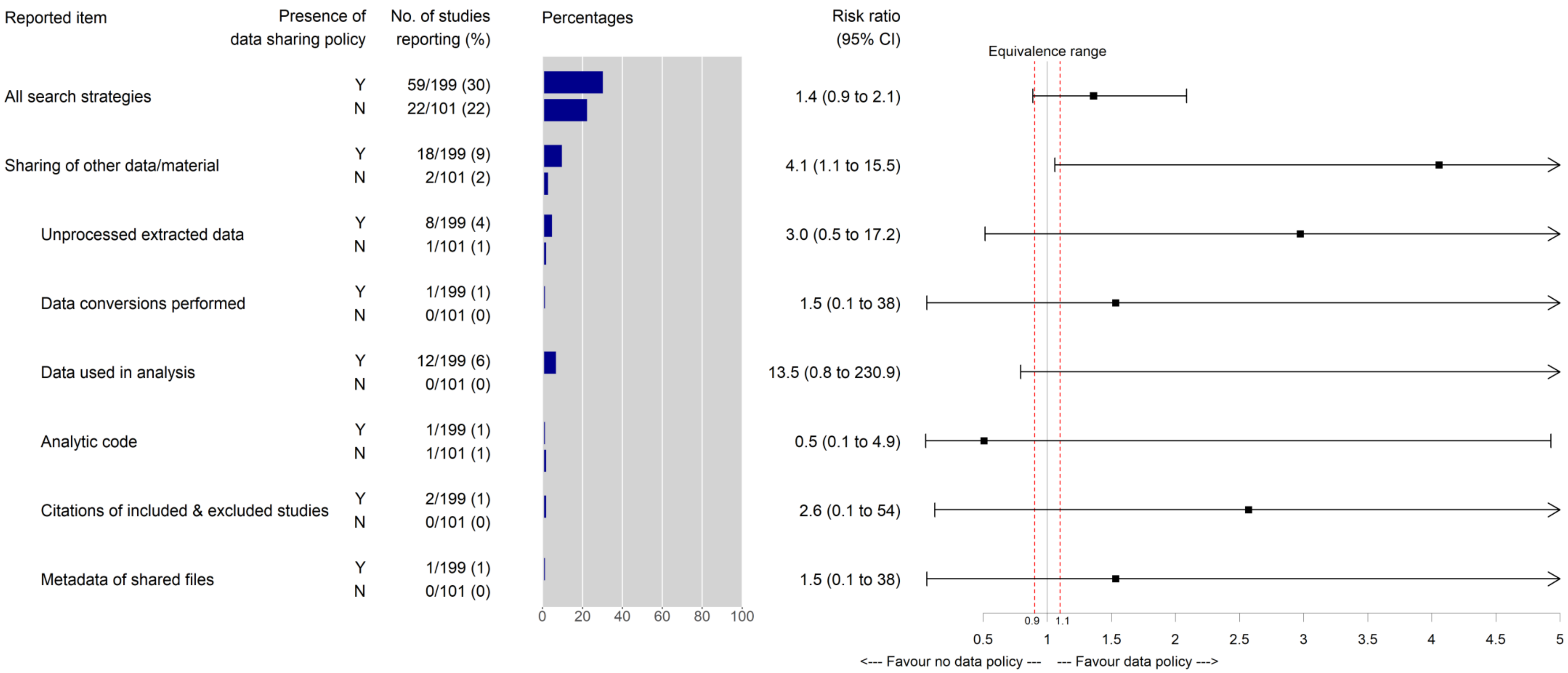

Supplement: Supplementary file 1 — Web appendix: Supplemental data [file ngup072428.ww.pdf]
